# Crystal structures of three 6-substituted coumarin-3-carboxamide derivatives

**DOI:** 10.1107/S2056989016008665

**Published:** 2016-06-10

**Authors:** Lígia R. Gomes, John Nicolson Low, André Fonseca, Maria João Matos, Fernanda Borges

**Affiliations:** a REQUIMTE, Departamento de Química e Bioquímica, Faculdade de Ciências da Universidade do Porto, Rua do Campo Alegre, 687, P-4169-007, Porto, Portugal; b FP-ENAS-Faculdade de Ciências de Saúde, Escola Superior de Saúde da UFP, Universidade Fernando Pessoa, Rua Carlos da Maia, 296, P-4200-150 Porto, Portugal; c Department of Chemistry, University of Aberdeen, Meston Walk, Old Aberdeen AB24 3UE, Scotland; d CIQUP/Departamento de Química e Bioquímica, Faculdade de Ciências, Universidade do Porto, 4169-007 Porto, Portugal

**Keywords:** crystal structure, coumarin, carboxamide

## Abstract

Three coumarin derivatives, *viz.* 6-methyl-*N*-(3-methyl­phen­yl)-2-oxo-2*H*-chromene-3-carboxamide, C_18_H_15_NO_3_ (**1**), *N*-(3-meth­oxy­phen­yl)-6-methyl-2-oxo-2*H*-chromene-3-carboxamide, C_18_H_15_NO_4_ (**2**), and 6-meth­oxy-*N*-(3-meth­oxy­phen­yl)-2-oxo-2*H*-chromene-3-carboxamide, C_18_H_15_NO_5_ (**3**), were synthesized and structurally characterized. The mol­ecules display intra­molecular N—H⋯O and weak C—H⋯O hydrogen bonds, which probably contribute to the approximate planarity of the mol­ecules. The supra­molecular structures feature C—H⋯O hydrogen bonds and π–π inter­actions, as confirmed by Hirshfeld surface analyses.

## Abstract 2

Three coumarin derivatives display intra­molecular N—H⋯O and weak C—H⋯O hydrogen bonds, which probably contribute to the approximate planarity of the mol­ecules. The supra­molecular structures feature C—H⋯O hydrogen bonds and π–π inter­actions, as confirmed by Hirshfeld surface analyses.

## Chemical context   

Benzopyrones are oxygen-containing heterocycles recognised as privileged structures for drug-discovery programs (Klekota & Roth, 2008 ▸; Lachance *et al.*, 2012 ▸). Within this class of compounds, coumarin has emerged as an inter­esting building block due to its synthetic accessibility and substitution variability. Furthermore, coumarins display anti­cancer, anti­viral, anti-inflammatory and anti-oxidant biological properties (Matos *et al.*, 2009 ▸, 2014 ▸; Vazquez-Rodriguez *et al.*, 2013 ▸).
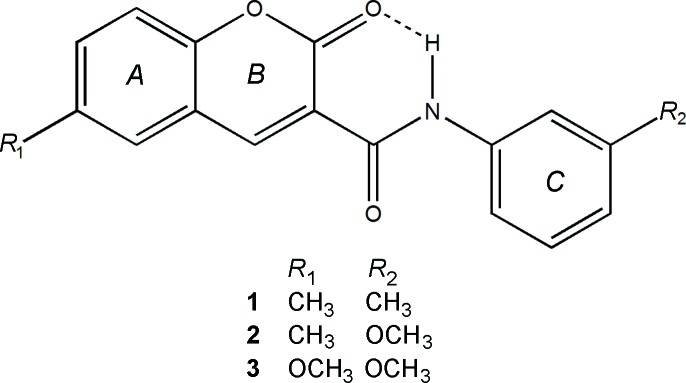


Previous work reported by our research group has shown that coumarin is a valid scaffold for the development of mono­amino oxidase B inhibitors (Matos *et al.*, 2009 ▸). As part of our ongoing studies of these compounds, we now describe the syntheses and crystal structures of three coumarin deriv­atives: 6-methyl-*N*-(3-methyl­phen­yl)-2-oxo-2*H*-chromene-3-carboxamide (**1**), *N*-(3-meth­oxy­phen­yl)-6-methyl-2-oxo-2*H*-chromene-3-carboxamide (**2**) and 6-meth­oxy-*N*-(3-meth­oxy­phen­yl)-2-oxo-2*H*-chromene-3-carboxamide (**3**).

## Structural commentary   

**Figure 1:**
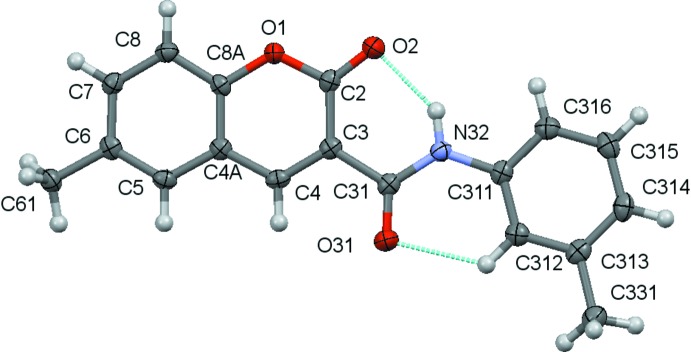
A view of the asymmetric unit of **1** with the atom-numbering scheme. Displacement ellipsoids are drawn at the 70% probability level.

The structural analyses revealed that the mol­ecules are coumarin derivatives with a phenyl­amide substituent at position 3 of the coumarin ring, as seen in the chemical scheme. The coumarin component rings are identified by the letters *A* and *B* while the exocyclic benzene ring is denoted *C*. Figs. 1 ▸–3 ▸ ▸ show the mol­ecular structures of compounds **1**–**3**, respectively: they differ in the type of substituents at the 6-position of the coumarin ring system and at the 3-position of the pendant benzene ring.

An inspection of the bond lengths shows that there is a slight asymmetry of the electronic distribution around the coumarin ring: the mean C3—C4 bond length [1.3517 (3) Å] and the mean value for the C3—C2 bond length [1.461 (6) Å)] are shorter and longer, respectively, that those expected for an C_*ar*_—C_*ar*_ bond, suggesting that there is an increased electronic density located in the C3—C4 bond at the pyrone ring.

The values for the distances of the C3—C31 bonds [mean value 1.508 (4) Å] connecting the coumarin system to the amide spacer are of the same order as a C*sp^3^*—C*sp^3^* bond. This confers freedom of rotation of the phenyl­amide substituent around it. Despite that, the mol­ecules are approximately planar, as can be inferred by the set of values of the dihedral angles in Table 1 ▸, which refer to the combination of the dihedral angles between the best planes formed by all non-H atoms of the 2*H*-chromen-2-one ring, the O31/C31/N32 atoms of the amide residue and the phenyl substituent, which are all less than 11°. This may be correlated with the conformation assumed by the amide group around the C—N rotamer which displays an −*anti* orientation with respect to the *oxo* oxygen atom of the coumarin, thus allowing the establishment, in all three structures, of an intra­molecular N—H⋯O hydrogen bond between the amino group of the carboxamide and the *oxo* group at the O2 position of the coumarin and a weak C—H⋯O intra­molecular hydrogen bond between an *ortho*-CH group on the exocyclic phenyl ring and the O atom of the carboxamide. Thus these two inter­actions, which both form *S*(6) rings, probably contribute to the overall approximate planarity of the mol­ecules since they may prevent the mol­ecules from adopting some other possible conformations by restraining their geometry.

## Supra­molecular features   

As mentioned above, the NH group is involved in an intra­molecular hydrogen bond. It is not involved in any inter­molecular inter­actions thus only carbon atoms may act as donors for the carbonyl and meth­oxy-type acceptors. Details of the hydrogen bonding for compounds **1**, **2** and **3** are given in Tables 2 ▸, 3 ▸ and 4 ▸, respectively.

**Figure 4:**
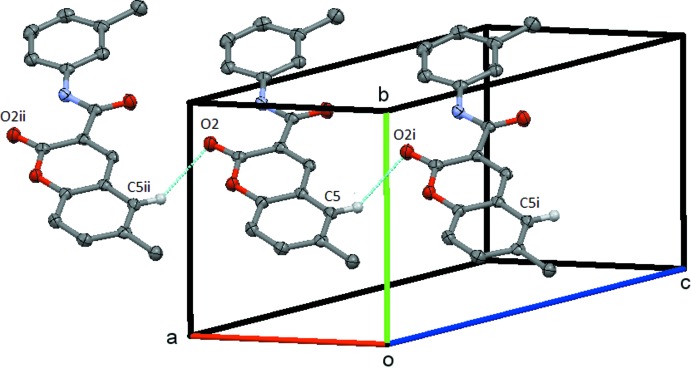
Compound **1**, the simple chain formed by the C5—H5⋯O1 weak hydrogen bond. This chain extends by unit translation along the *a* axis. Symmetry codes: (i) *x* − 1, *y*, *z;* (ii) *x* + 1, *y*, *z*. H atoms not involved in the hydrogen bonding are omitted.

**Figure 5:**
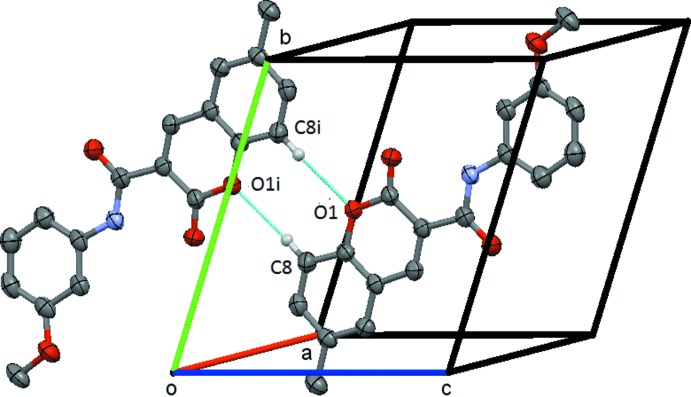
Compound **2**, view of the C8—H8⋯O1 centrosymmetric 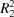
(8) ring structure centred on (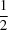, 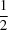, 0). Symmetry code: (i) −*x* + 1, −*y* + 1, *z*. H atoms not involved in the hydrogen bonding are omitted.

**Figure 6:**
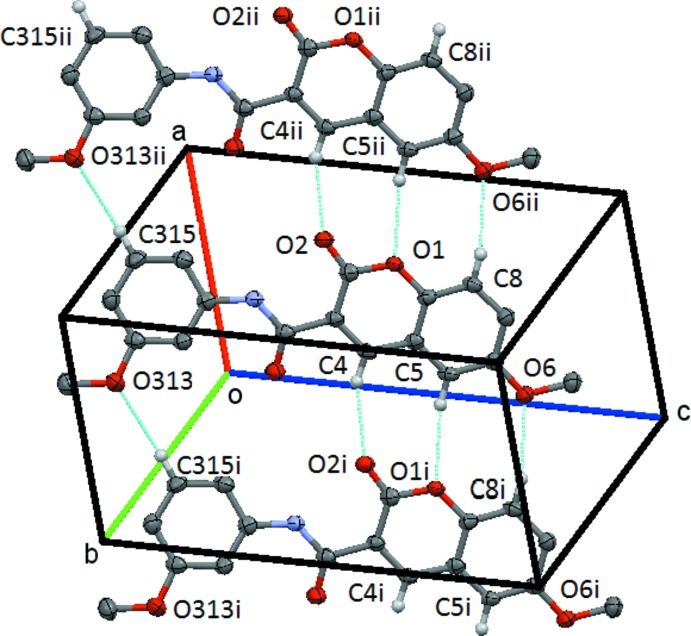
Compound **3**, view of the chain of the linked 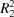
(8), 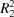
(8) and 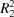
(16) structures formed by the inter­action of the C8—H8⋯O6, C5—H5⋯O1, C4—H4⋯O1 and C315—H315⋯O313 hydrogen bonds. This chain extends by unit translation along the *a* axis. Symmetry codes: (i) *x* − 1, *y*, *z*; (ii) −*x* + 1, *y*, *z*. H atoms not involved in the hydrogen bonding are omitted.

In **1**, the mol­ecules are linked by the C5—H5⋯O1(*x* − 1, *y*, *z*) weak hydrogen bond to form a *C*(6) chain, which runs parallel to the *a* axis, Fig. 4 ▸. In **2**, the mol­ecules are linked by the C8—H8⋯O1(−*x* + 1, −*y* + 1, −*z*) weak hydrogen bond to form an 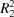
(8) centrosymmetric dimer centred on (1/2, 1/2, 0), Fig. 5 ▸. There is also a short C317—H31*A*⋯O31(*x*, *y* + 1, *z*) contact involving a methyl hydrogen atom. In **3**, the mol­ecules are linked by the C4—H4⋯O2(*x* − 1, *y*, *z*), C5—H5⋯O1(*x* − 1, *y*, *z*) and C8—H8⋯O6(*x* + 1, *y*, *z*) bonds to form a chain of 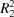
(8) rings, which runs parallel to the *a* axis, Fig. 6 ▸. This chain is supplemented by the action of the C315—H315⋯O313(*x* + 1, *y*, *z*) weak hydrogen bond.

## Hirshfeld surfaces   

**Figure 7:**
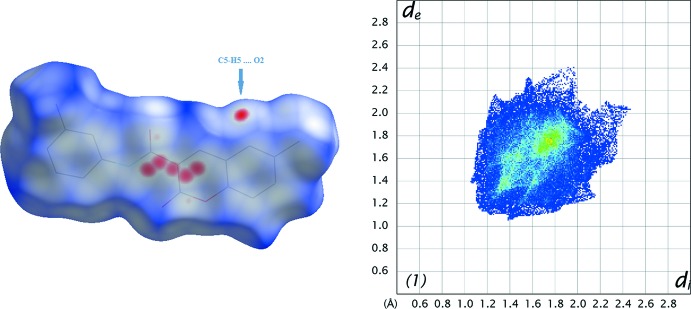
A view of the Hirshfeld surface mapped over *d*_norm_ (left) and fingerprint plot (right) for **1**. The highlighted red spots on the top face of the surfaces indicate contact points with the atoms participating in the C—H⋯O inter­molecular inter­actions whereas those on the middle of the surface correspond to C⋯C contacts consequent of the π–π stacking. The C⋯C contacts contribute to higher the frequency of the pixels at *d*_e_ ≃ *d*_i_ ≃ 1.8 Å on the FP plots (yellow spot).

The Hirshfeld surfaces and two-dimensional fingerprint (FP) plots (Rohl *et al.*, 2008 ▸) were generated using *Crystal Explorer 3.1* (Wolff *et al.*, 2012 ▸). The surfaces, mapped over *d*_norm_ and the FP plots are presented in Figs. 7 ▸ to 9 ▸ ▸ for **1**, **2** and **3**, respectively. They provide complementary information concerning the inter­molecular inter­actions discussed above. The contributions from various contacts, listed in Table 5 ▸, were selected by the partial analysis of the FP plots.

Forgetting the prevalence of the H⋯H contacts on the surface, inherent to organic mol­ecules, the most significant contacts are the H⋯O/O⋯H ones. Those appear as highlighted red spots on the top face of the surfaces (Fig. 7 ▸ to 9) that indicate contact points with the atoms participating in the C—H⋯O inter­molecular inter­actions. Those contacts corres­pond to weak hydrogen bonds, as seen in the FP plots where the pair of sharp spikes that would be characteristic of hydrogen bond are masked by the H⋯H inter­actions appearing near *d*_e_≃*d*_i_ = 1.20 Å. Compound **1** has the smallest percentage for H⋯O/O⋯H contacts since it has no meth­oxy substituents. The most representative of these corresponds to the C5—H5⋯O2 contact that links the mol­ecules in the C6 chain. In the surface of **2**, two red spots appear perpendicular to the C8—H8 bond and near O1 indicating the C8—H8⋯O1 contact that links the mol­ecules into dimers. The red spots near O31 indicate that this atom establishes two weak contacts (C61—H61*B*⋯O31 and C317—H31*A*⋯O31). In **3**, there are several contacts, three of those involving the oxygen atoms of the coumarin system and those directly connected to it that are acceptors for H atoms of the coumarin residue of another mol­ecule. These multiple contacts result in chains of hydrogen-bonded rings, as described in the previous section, and seem to operate a co-operative effect since the hydrogen bonds in **3** are stronger than in **1** and **2** (see the well-defined sharp spikes in the FP plot of **3**).

**Figure 10:**
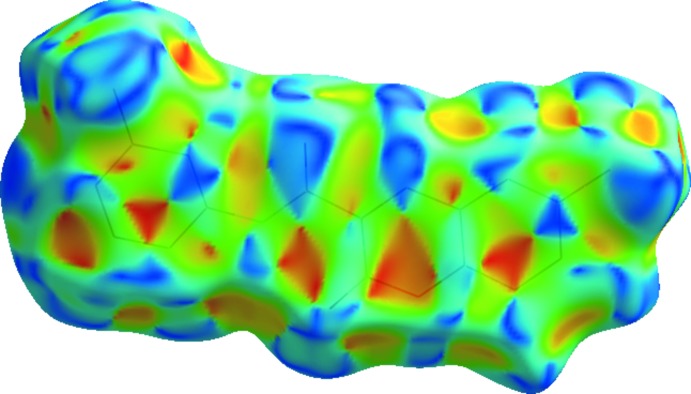
Surface of **1** mapped with shape index showing the complementary triangular red hollows and blue bumps that are characteristic of six-ring stacking.

The values for the remaining contacts listed in Table 5 ▸ suggest that the supra­molecular structure is built by H⋯C/C⋯H and C⋯C contacts. In **3**, the percentage for H⋯C/C⋯H contacts is higher than that for the other compounds. The FP plots also reveal a cluster at *d*_e_/*d*_i_ ≃ 1.8 Å and *d*_i_/*d*_e_ ≃ 1.2 Å characteristic of C—H⋯π contacts that seem to assume higher importance in the supra­molecular structure in **3**. On the other hand, the C⋯C contacts prevail in **1** and **2**. In fact, the packing in **1** is built up by several π–π inter­actions (Table 6 ▸). Also, when the surface is mapped with shape index, several complementary triangular red hollows and blue bumps appear that are characteristic of the six-ring stacking (Figs. 10 ▸ and 11 ▸). In **1**, ring *A* stacks with ring *C* by a twofold rotation, and ring *B* with ring *A* when the mol­ecule is placed above another centrosymmetrically related mol­ecule. This gives rise to close C⋯C contacts in the middle of the surface identified as red spots. Mol­ecule **2** also displays a significant percentage of C⋯C contacts on the Hirshfeld surface, resulting from the continuous π–π stacking where ring *C* stacks with rings *A* and *B* (up and down) of centrosymmetrically related mol­ecules.

## Database survey   

A search made in the Cambridge Structural Database (Groom *et al.*, 2016 ▸) revealed the existence of 35 deposited compounds (42 mol­ecules) containing the coumarin carboxamide unit, all of which contained the same intra­molecular N—H⋯O hydrogen bond as seen here. The hydrogen atoms in these structures were riding with ideally fixed positions or refined positions. The range of values for N—H were 0.78 to 1.02 Å with a median value of 0.88 Å, the range of values for H⋯O were 1.87 to 2.04 Å with a median value of 2.00 Å, the range of values for N⋯O were 2.639 to 2.801 Å with a median value of 2.722 Å and the range of values for the N—H⋯O angle was 125 to 146° with a median value of 138°.

Six of these compounds, with CSD codes: BONKAS (Julien *et al.*, 2014 ▸); DISXUA, DISYAH, DISYEL and DISYIP (Maldonado-Domínguez *et al.*, 2014 ▸); WOJXOK (Pan *et al.*, 2014 ▸), have a phenyl group attached to the carboxamide N atom and these mol­ecules have similar conformations to the present compounds, Table 1 ▸. These compounds also had a short intra­molecular contact between the *ortho-*C hydrogen atom of the exocyclic benzene ring and the carboxamide O atom as in the present compounds. Details of the searches can be found in the supporting information.

## Synthesis and crystallization   

The coumarin derivatives **1**–**3** were synthesized by a two-step process. In the first step, 5-methyl­salicyl­aldehyde (1 mmol) and diethyl malonate (1 mmol) and catalytic amounts of piperidine were dissolved in ethanol (10 ml) and refluxed for 4 h. After cooling to room temperature, the suspension was filtered off and ethyl 6-methyl­coumarin-3-carboxyl­ate was obtained. This compound was then dissolved in 20 ml of an ethano­lic solution with 0.5% NaOH (aq.) and hydrolyzed under reflux for 1h. After reaction, 10% HCl (aq.) was added and the desired carb­oxy­lic acid was then filtered and washed with water (Chimenti *et al.*, 2010 ▸).

Then, to a solution of 6-methyl­coumarin-3-carb­oxy­lic acid (1 mmol) in di­chloro­methane, 1-ethyl-3-(3-di­methyl­amino­prop­yl)carbodi­imide (EDC) (1.10 mmol) and 4-di­methyl­amino­pyridine (DMAP) (1.10 mmol) were added. The mixture was kept under a flux of argon gas at 273 K for five minutes. Shortly after, the aromatic amine (1 mmol) with the intended substitution pattern was added. The reaction mixture was stirred for 4 h at room temperature. The crude product was filtered and purified by column chromatography (hexa­ne/ethyl acetate 9:1) or by recrystallization with ethanol to give the desired product, (Murata *et al.*, 2005 ▸). 6-Methyl-*N*-(3′-methyl­phen­yl)coumarin-3-carboxamide (**1**) (yield: 79%; m.p. 467–468 K; crystallization solvent: methanol); 6-methyl-*N*-(3′-meth­oxy­phen­yl)coumarin-3-carboxamide (**2**) (yield: 74%; m.p. 447–448 K; crystallization solvent: methanol); 6-meth­oxy-*N*-(3′-meth­oxy­phen­yl)coumarin-3-carboxamide (**3**) (yield: 50.7%; m.p. 440–441 K; crystallization solvent: ethyl acetate).

## Refinement   

Crystal data, data collection and structure refinement details are summarized in Table 7 ▸. H atoms were treated as riding atoms with C—H(aromatic) = 0.95 Å and *U*_iso_ = 1.2*U*_eq_(C), C—H(meth­yl) 0.98 Å and *U*_iso_ = 1.5*U*_eq_(C) The amino H atoms were refined.

## Supplementary Material

Crystal structure: contains datablock(s) 1, 2, general, 3. DOI: 10.1107/S2056989016008665/hb7589sup1.cif

Structure factors: contains datablock(s) 1. DOI: 10.1107/S2056989016008665/hb75891sup2.hkl

Structure factors: contains datablock(s) 2. DOI: 10.1107/S2056989016008665/hb75892sup3.hkl

Structure factors: contains datablock(s) 3. DOI: 10.1107/S2056989016008665/hb75893sup4.hkl

Supporting information file. DOI: 10.1107/S2056989016008665/hb75891sup5.cml

Supporting information file. DOI: 10.1107/S2056989016008665/hb75892sup6.cml

Supporting information file. DOI: 10.1107/S2056989016008665/hb75893sup7.cml

Supporting information file. DOI: 10.1107/S2056989016008665/hb7589sup8.pdf

Supporting information file. DOI: 10.1107/S2056989016008665/hb7589sup9.pdf

CCDC references: 1482450, 1482449, 1482448

Additional supporting information:  crystallographic information; 3D view; checkCIF report

## Figures and Tables

**Figure 2:**
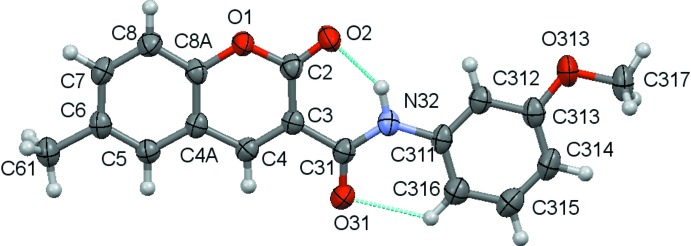
A view of the asymmetric unit of **2** with the atom-numbering scheme. Displacement ellipsoids are drawn at the 70% probability level.

**Figure 3:**
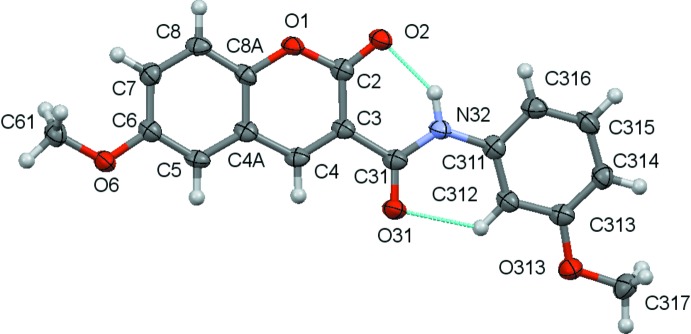
A view of the asymmetric unit of **3** with the atom-numbering scheme. Displacement ellipsoids are drawn at the 70% probability level.

**Figure 8:**
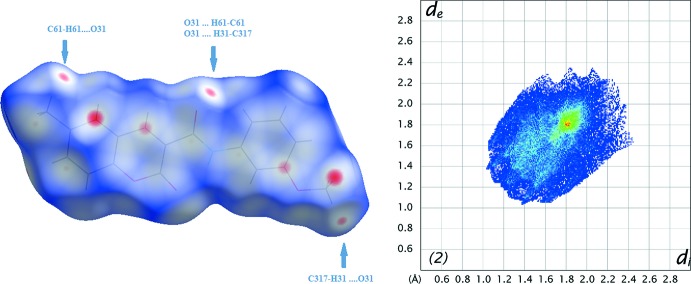
A view of the Hirshfeld surface mapped over *d*_norm_ (left) and fingerprint plot (right) for **2**. The highlighted red spots on the top face of the surfaces indicate contact points with the atoms participating in the C–H⋯O inter­molecular inter­actions whereas those on the middle of the surface correspond to C⋯C contacts consequent of the π–π stacking. The C⋯C contacts contribute to higher the frequency of the pixels at *d*_e_ ≃ *d*_i_ ≃ 1.8 Å on the FP plots.

**Figure 9:**
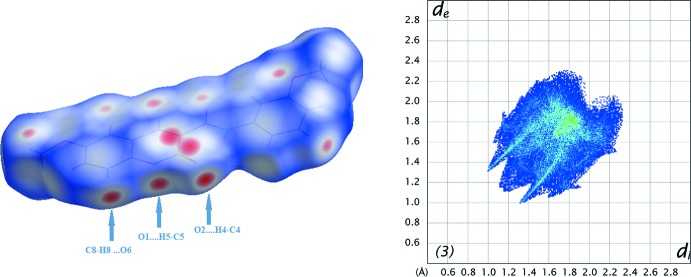
A view of the Hirshfeld surface mapped over *d*_norm_ (left) and fingerprint plot (right) for **3**. The highlighted red spots on the bottom face of the surfaces indicate contact points with the atoms participating in the C—H⋯O inter­molecular inter­actions whereas those on the middle of the surface correspond to C⋯C and C⋯H contacts. The FP plot displays two couple of spikes (external ends corresponding to C⋯H contacts and middle spikes corresponding to O⋯H contacts).

**Figure 11:**
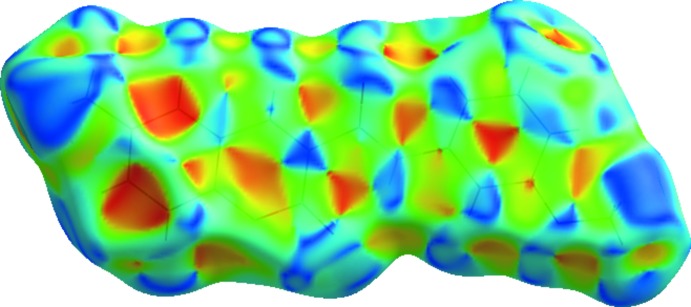
Surface of **2** mapped with shape index showing the complementary triangular red hollows and blue bumps that are characteristic of six-ring stacking.

**Table 1:** Selected dihedral angles (°) θ_1_ is the dihedral angle between the mean planes of the coumarin ring system and exocyclic phenyl ring. θ_2_ is the dihedral angles between the mean plane of the coumarin ring system and the plane defined by the atoms O31/C31/N32. θ_3_ is the dihedral angle between the mean planes of the exocyclic phenyl ring and the plane defined by atoms O31/C31/N32.

Compound	θ_1_	θ_2_	θ_3_
**1**	4.69 (6)	4.8 (2)	0.21 (23)
**2**	4.28 (3)	4.46 (13)	8.60 (12)
**3**	8.17 (13)	2.9 (4)	10.2 (4)
BONKAS	4.70 (6)	3.2 (2)	7.8 (2)
DISXUA	10.29 (7)	3.9 (2)	6.42)
DISYAH	0.04 (6)	2.70 (17)’	2.76 (17)
DISYEL	3.07 (8)	3.4 (2)	1.0 (3)
DISYIP	12.75 (6)	1.21 (17)	12.73 (17)
WOJXOK	1.9 (4)	4.6 (9)	2.7 (9)

If the mean planes for the combined coumarin ring system and exocyclic phenyl rings are considered, then the maximum deviations of atoms within these rings from this plane are −0,1024 (12) Å or C6 in **1**, −0.0754 (15) Å in **2** and 0.0699 (14) Å in **3**. Considering all non-hydrogen atoms, the maximum deviations from this plane are 0.1783 (10) Å for O31 in **1**, −0.1809 (12) Å for O31 in **2** and −0.2181 (15) Å for O313 in **3**.

**Table 2:** Hydrogen-bond geometry (Å, °) for **1** [Chem scheme1]

*D*—H⋯*A*	*D*—H	H⋯*A*	*D*⋯*A*	*D*—H⋯*A*
N32—H32⋯O2	0.893 (18)	1.957 (18)	2.7149 (14)	141.7 (16)
C312—H312⋯O31	0.95	2.26	2.8838 (16)	122
C5—H5⋯O1^i^	0.95	2.98	3.7304 (15)	137

Symmetry code: (i) 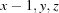.

**Table 3:** Hydrogen-bond geometry (Å, °) for **2** [Chem scheme1]

*D*—H⋯*A*	*D*—H	H⋯*A*	*D*⋯*A*	*D*—H⋯*A*
N32—H32⋯O2	0.96 (2)	1.85 (2)	2.6952 (16)	145.7 (17)
C8—H8⋯O1^i^	0.95	2.52	3.3676 (18)	149
C61—H61*B*⋯O31^ii^	0.98	2.57	3.4044 (19)	143
C317—H31*A*⋯O31^iii^	0.98	2.57	3.2769 (19)	129

Symmetry codes: (i) 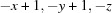; (ii) 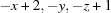; (iii) 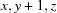.

**Table 4:** Hydrogen-bond geometry (Å, °) for **3** [Chem scheme1]

*D*—H⋯*A*	*D*—H	H⋯*A*	*D*⋯*A*	*D*—H⋯*A*
N32—H32⋯O2	0.92 (3)	1.91 (3)	2.699 (2)	143 (2)
C4—H4⋯O2^i^	0.95	2.43	3.319 (3)	155
C5—H5⋯O1^i^	0.95	2.47	3.391 (3)	164
C8—H8⋯O6^ii^	0.95	2.46	3.364 (3)	160
C312—H312⋯O31	0.95	2.26	2.868 (3)	121
C315—H315⋯O313^ii^	0.95	2.59	3.536 (4)	171

Symmetry codes: (i) 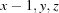; (ii) 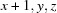.

**Table 5:** Percentages of atom–atom contacts

Contact	**1**	**2**	**3**
H⋯H	47.1	42.9	38.3
H⋯O/O⋯H	19.9	26.9	27.4
H⋯C/C⋯H	14.5	12.9	20.7
H⋯N/N⋯H	1.5	0.2	1.6
C⋯C	12.1	12.6	5.4

**Table 6:** Selected π–π contacts (Å) *CgI*(*J*) = plane number *I*(*J*); *Cg*⋯*Cg* = distance between ring centroids; *Cg*I_perp_ = perpendicular distance of *Cg*(*I*) on ring *J*; *CgJ*_perp_ = perpendicular distance of *Cg*(*J*) on ring *I*; Slippage = distance between *Cg*(I) and perpendicular projection of *Cg*(*J*) on ring *I*.

Compound	*Cg*I	*CgJ*(aru)	*Cg*⋯*Cg*	*CgI*_perp_	*CgJ*_perp_	Slippage
**1**	*Cg*1	*Cg*1(−*x* + 1, −*y* + 1, −*z*)	3.7630 (7)	−3.3400 (5)	−3.3400 (5)	1.733
**1**	*Cg*1	*Cg*2(−*x* + 1, −*y* + 1, −*z*)	3.4853 (7)	−3.3281 (5)	−3.3171 (5)	1.069
**1**	*Cg*2	*Cg*1(−*x* + 1, −*y* + 1, −*z*)	3.4853 (7)	−3.3172 (5)	−3.3281 (5)	1.035
**1**	*Cg*2	*Cg*3(−*x* + 1, −*y* + 2, −*z*)	3.6253 (7)	3.3547 (5)	3.4673 (5)	1.058
**1**	*Cg*3	*Cg*2(−*x* + 1, −*y* + 1, −*z*)	3.6253 (7)	3.4673 (5)	3.3548 (5)	1.374
						
**2**	*Cg*1	*Cg*3(−*x* + 1, −*y* + 1, −*z* + 1)	3.5379 (9)	−3.4691 (6)	−3.4872 (6)	0.597
**2**	*Cg*3	*Cg*1(−*x* + 1, −*y* + 1, −*z* + 1)	3.5378 (9)	−3.4872 (6)	−3.4691 (6)	0.694
**2**	*Cg*1	*Cg*3(−*x* + 2, −*y* + 1, −*z* + 1)	3.5974 (9)	3.4237 (6)	3.4068 (6)	1.156
**2**	*Cg*3	*Cg*1(−*x* + 2, −*y* + 1, −*z* + 1)	3.5975 (9)	3.4069 (6)	3.4237 (6)	1.105
**2**	*Cg*2	*Cg*3(−*x* + 1, −*y* + 1, −*z* + 1)	3.9325 (9)	−3.5309 (6)	−3.4844 (6)	1.823
**2**	*Cg*3	*Cg*2(−*x* + 1, −*y* + 1, −*z* + 1)	3.9324 (9)	−3.4844 (6)	−3.5309 (6)	1.731
						
**3**	*Cg*1	*Cg*2(−*x* + 1, −*y*, −*z* + 1)	3.5978 (13)	−3.3575 (9)	−3.3307 (9)	1.360
**3**	*Cg*2	*Cg*1(−*x* + 1, −*y*, −*z* + 1)	3.5978 (13)	−3.3307 (9)	−3.3575 (9)	1.293

Plane 1 is the plane of the pyran ring with *Cg*1 as centroid, ring *B*. Plane 2 is the plane of the coumarin phenyl ring with *Cg*2 as centroid, ring *A*. Plane 3 is the plane of the exocyclic phenyl ring with *Cg*3 as centroid, ring *C*. Some planes are repeated since they are inclined to each other and as a result give slightly different slippages.

**Table 7:** Experimental details

	**1**	**2**	**3**
Crystal data			
Chemical formula	C_18_H_15_NO_3_	C_18_H_15_NO_4_	C_18_H_15_NO_5_
*M*_r_	293.31	309.31	325.31
Crystal system, space group	Monoclinic, *P*2_1_/*c*	Triclinic, *P* 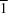	Triclinic, *P* 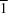
Temperature (K)	100	100	100
*a*, *b*, *c* (Å)	7.2117 (3), 8.0491 (3), 23.6242 (9)	7.1028 (4), 10.1367 (4), 10.8171 (5)	6.7722 (5), 8.3098 (7), 14.4202 (13)
α, β, γ (°)	90, 94.388 (4), 90	75.827 (4), 88.318 (4), 71.271 (4)	91.874 (7), 100.009 (7), 113.042 (7)
*V* (Å^3^)	1367.31 (9)	714.10 (6)	730.84 (11)
*Z*	4	2	2
Radiation type	Mo *K*α	Mo *K*α	Mo *K*α
μ (mm^−1^)	0.10	0.10	0.11
Crystal size (mm)	0.42 × 0.03 × 0.02	0.20 × 0.04 × 0.02	0.17 × 0.11 × 0.02

Data collection			
Diffractometer	Rigaku AFC12 (Right)	Rigaku AFC12 (Right)	Rigaku AFC12 (Right)
Absorption correction	Multi-scan (*CrysAlis PRO*; Rigaku Oxford Diffraction, 2015 ▸)	Multi-scan (*CrysAlis PRO*; Rigaku Oxford Diffraction, 2015 ▸)	Multi-scan (*CrysAlis PRO*; Rigaku Oxford Diffraction, 2015 ▸)
*T*_min_, *T*_max_	0.895, 1.000	0.893, 1.000	0.792, 1.000
No. of measured, independent and observed [*I* > 2σ(*I*)] reflections	12045, 3135, 2593	15638, 3262, 2704	8745, 3302, 2666
*R*_int_	0.023	0.025	0.033
(sin θ/λ)_max_ (Å^−1^)	0.649	0.649	0.649

Refinement			
*R*[*F* ^2^ > 2σ(*F* ^2^)], *wR*(*F* ^2^), *S*	0.041, 0.120, 1.03	0.047, 0.139, 1.02	0.071, 0.152, 1.16
No. of reflections	3134	3261	3302
No. of parameters	205	214	223
H-atom treatment	H atoms treated by a mixture of independent and constrained refinement	H atoms treated by a mixture of independent and constrained refinement	H atoms treated by a mixture of independent and constrained refinement
Δρ_max_, Δρ_min_ (e Å^−3^)	0.35, −0.26	0.37, −0.21	0.25, −0.26

Computer programs: *CrysAlis PRO* (Rigaku Oxford Diffraction, 2015 ▸), *OSCAIL* (McArdle *et al.*, 2004 ▸), *SHELXT* (Sheldrick, 2015*a* ▸), *ShelXle* (Hübschle *et al.*, 2011 ▸), *SHELXL2014* (Sheldrick, 2015*b* ▸), *Mercury* (Macrae *et al.*, 2006 ▸) and *PLATON* (Spek, 2009 ▸).

## supplementary crystallographic information

### (1) 6-Methyl-N-(3-methylphenyl)-2-oxo-2H-chromene-3-carboxamide Crystal data

**Table:**

C_18_H_15_NO_3_	*F*(000) = 616
*M**_r_* = 293.31	*D*_x_ = 1.425 Mg m^−^^3^
Monoclinic, *P*2_1_/*c*	Mo *K*α radiation, λ = 0.71075 Å
*a* = 7.2117 (3) Å	Cell parameters from 5809 reflections
*b* = 8.0491 (3) Å	θ = 2.7–27.6°
*c* = 23.6242 (9) Å	µ = 0.10 mm^−^^1^
β = 94.388 (4)°	*T* = 100 K
*V* = 1367.31 (9) Å^3^	Needle, yellow
*Z* = 4	0.42 × 0.03 × 0.02 mm

### (1) 6-Methyl-N-(3-methylphenyl)-2-oxo-2H-chromene-3-carboxamide Data collection

**Table:**

Rigaku AFC12 (Right) diffractometer	3135 independent reflections
Radiation source: Rotating Anode	2593 reflections with *I* > 2σ(*I*)
Detector resolution: 28.5714 pixels mm^-1^	*R*_int_ = 0.023
profile data from ω–scans	θ_max_ = 27.5°, θ_min_ = 1.7°
Absorption correction: multi-scan (CrysAlis PRO; Rigaku Oxford Diffraction, 2015)	*h* = −9→8
*T*_min_ = 0.895, *T*_max_ = 1.000	*k* = −10→7
12045 measured reflections	*l* = −30→29

### (1) 6-Methyl-N-(3-methylphenyl)-2-oxo-2H-chromene-3-carboxamide Refinement

**Table:**

Refinement on *F*^2^	0 restraints
Least-squares matrix: full	Hydrogen site location: mixed
*R*[*F*^2^ > 2σ(*F*^2^)] = 0.041	H atoms treated by a mixture of independent and constrained refinement
*wR*(*F*^2^) = 0.120	*w* = 1/[σ^2^(*F*_o_^2^) + (0.0741*P*)^2^ + 0.3348*P*] where *P* = (*F*_o_^2^ + 2*F*_c_^2^)/3
*S* = 1.03	(Δ/σ)_max_ = 0.001
3134 reflections	Δρ_max_ = 0.35 e Å^−^^3^
205 parameters	Δρ_min_ = −0.26 e Å^−^^3^

### (1) 6-Methyl-N-(3-methylphenyl)-2-oxo-2H-chromene-3-carboxamide Special details

**Table:**

Experimental. CrysAlisPro 1.171.38.41 (Rigaku Oxford Diffraction, 2015) Empirical absorption correction using spherical harmonics, implemented in SCALE3 ABSPACK scaling algorithm.
Geometry. All esds (except the esd in the dihedral angle between two l.s. planes) are estimated using the full covariance matrix. The cell esds are taken into account individually in the estimation of esds in distances, angles and torsion angles; correlations between esds in cell parameters are only used when they are defined by crystal symmetry. An approximate (isotropic) treatment of cell esds is used for estimating esds involving l.s. planes.

### (1) 6-Methyl-N-(3-methylphenyl)-2-oxo-2H-chromene-3-carboxamide Fractional atomic coordinates and isotropic or equivalent isotropic displacement parameters (Å^2^)

**Table:**

	*x*	*y*	*z*	*U*_iso_*/*U*_eq_	
O1	0.69397 (12)	0.68051 (12)	−0.05947 (3)	0.0158 (2)	
O2	0.88820 (13)	0.82921 (12)	−0.00415 (4)	0.0191 (2)	
O31	0.45616 (13)	0.95381 (12)	0.10022 (4)	0.0199 (2)	
N32	0.76928 (15)	0.97441 (14)	0.09028 (4)	0.0153 (2)	
H32	0.855 (2)	0.949 (2)	0.0664 (7)	0.033 (5)*	
C2	0.72934 (18)	0.77998 (16)	−0.01268 (5)	0.0147 (3)	
C3	0.57335 (17)	0.81575 (16)	0.02182 (5)	0.0137 (3)	
C4	0.40358 (17)	0.75122 (16)	0.00695 (5)	0.0144 (3)	
H4	0.3034	0.7759	0.0294	0.017*	
C4A	0.37082 (17)	0.64663 (15)	−0.04177 (5)	0.0140 (3)	
C5	0.19774 (18)	0.57410 (16)	−0.05816 (5)	0.0152 (3)	
H5	0.0946	0.5956	−0.0365	0.018*	
C6	0.17503 (18)	0.47192 (16)	−0.10530 (5)	0.0148 (3)	
C7	0.32959 (18)	0.44378 (16)	−0.13696 (5)	0.0159 (3)	
H7	0.3155	0.3739	−0.1694	0.019*	
C8	0.50106 (18)	0.51447 (16)	−0.12233 (5)	0.0160 (3)	
H8	0.6034	0.4950	−0.1445	0.019*	
C8A	0.52020 (17)	0.61462 (16)	−0.07444 (5)	0.0139 (3)	
C31	0.59396 (18)	0.92182 (16)	0.07469 (5)	0.0144 (3)	
C61	−0.01018 (18)	0.39458 (17)	−0.12285 (5)	0.0178 (3)	
H61A	0.0066	0.2759	−0.1302	0.027*	
H61B	−0.0642	0.4489	−0.1574	0.027*	
H61C	−0.0937	0.4085	−0.0924	0.027*	
C311	0.83193 (18)	1.06916 (16)	0.13848 (5)	0.0150 (3)	
C312	0.71473 (18)	1.12797 (16)	0.17828 (5)	0.0163 (3)	
H312	0.5861	1.1013	0.1745	0.020*	
C313	0.78614 (19)	1.22628 (17)	0.22372 (5)	0.0175 (3)	
C314	0.97525 (19)	1.26281 (17)	0.22921 (5)	0.0194 (3)	
H314	1.0244	1.3307	0.2597	0.023*	
C315	1.09272 (19)	1.20017 (17)	0.19020 (5)	0.0197 (3)	
H315	1.2221	1.2236	0.1947	0.024*	
C316	1.02253 (18)	1.10408 (17)	0.14486 (5)	0.0175 (3)	
H316	1.1032	1.0622	0.1183	0.021*	
C317	0.6556 (2)	1.29639 (18)	0.26456 (5)	0.0220 (3)	
H31A	0.7234	1.3144	0.3016	0.033*	
H31B	0.5535	1.2180	0.2687	0.033*	
H31C	0.6050	1.4023	0.2499	0.033*	

### (1) 6-Methyl-N-(3-methylphenyl)-2-oxo-2H-chromene-3-carboxamide Atomic displacement parameters (Å^2^)

**Table:**

	*U*^11^	*U*^22^	*U*^33^	*U*^12^	*U*^13^	*U*^23^
O1	0.0138 (5)	0.0177 (5)	0.0158 (4)	−0.0013 (4)	0.0014 (3)	−0.0033 (4)
O2	0.0158 (5)	0.0214 (5)	0.0204 (5)	−0.0018 (4)	0.0029 (3)	−0.0037 (4)
O31	0.0178 (5)	0.0229 (5)	0.0195 (5)	0.0001 (4)	0.0044 (4)	−0.0047 (4)
N32	0.0157 (6)	0.0153 (6)	0.0150 (5)	0.0015 (4)	0.0017 (4)	−0.0020 (4)
C2	0.0174 (7)	0.0116 (6)	0.0148 (6)	0.0008 (5)	0.0000 (5)	0.0004 (5)
C3	0.0166 (6)	0.0111 (6)	0.0136 (6)	0.0019 (5)	0.0013 (4)	0.0019 (5)
C4	0.0163 (6)	0.0122 (6)	0.0149 (6)	0.0036 (5)	0.0035 (5)	0.0021 (5)
C4A	0.0156 (6)	0.0115 (6)	0.0147 (6)	0.0021 (5)	0.0003 (5)	0.0020 (5)
C5	0.0143 (6)	0.0137 (6)	0.0179 (6)	0.0021 (5)	0.0027 (5)	0.0018 (5)
C6	0.0150 (6)	0.0126 (6)	0.0164 (6)	0.0022 (5)	−0.0016 (4)	0.0037 (5)
C7	0.0178 (7)	0.0157 (7)	0.0140 (6)	0.0013 (5)	−0.0006 (5)	−0.0010 (5)
C8	0.0153 (6)	0.0176 (7)	0.0154 (6)	0.0031 (5)	0.0025 (5)	0.0003 (5)
C8A	0.0127 (6)	0.0127 (6)	0.0161 (6)	0.0006 (5)	−0.0007 (5)	0.0021 (5)
C31	0.0179 (7)	0.0115 (6)	0.0138 (6)	0.0010 (5)	0.0012 (5)	0.0016 (5)
C61	0.0147 (6)	0.0175 (7)	0.0210 (6)	−0.0002 (5)	−0.0004 (5)	−0.0007 (5)
C311	0.0188 (7)	0.0116 (6)	0.0142 (6)	0.0011 (5)	−0.0005 (5)	0.0019 (5)
C312	0.0171 (6)	0.0152 (6)	0.0166 (6)	0.0016 (5)	0.0010 (5)	0.0025 (5)
C313	0.0237 (7)	0.0143 (7)	0.0147 (6)	0.0017 (5)	0.0020 (5)	0.0027 (5)
C314	0.0243 (7)	0.0168 (7)	0.0163 (6)	−0.0022 (5)	−0.0034 (5)	0.0010 (5)
C315	0.0180 (7)	0.0195 (7)	0.0210 (6)	−0.0025 (5)	−0.0020 (5)	0.0039 (5)
C316	0.0187 (7)	0.0172 (7)	0.0168 (6)	0.0014 (5)	0.0027 (5)	0.0032 (5)
C317	0.0247 (7)	0.0229 (7)	0.0183 (6)	0.0008 (6)	0.0018 (5)	−0.0026 (5)

### (1) 6-Methyl-N-(3-methylphenyl)-2-oxo-2H-chromene-3-carboxamide Geometric parameters (Å, º)

**Table:**

O1—C2	1.3730 (15)	C8—C8A	1.3874 (17)
O1—C8A	1.3817 (15)	C8—H8	0.9500
O2—C2	1.2144 (15)	C61—H61A	0.9800
O31—C31	1.2287 (15)	C61—H61B	0.9800
N32—C31	1.3573 (16)	C61—H61C	0.9800
N32—C311	1.4154 (16)	C311—C312	1.3943 (17)
N32—H32	0.893 (18)	C311—C316	1.3998 (18)
C2—C3	1.4672 (17)	C312—C313	1.3997 (18)
C3—C4	1.3514 (18)	C312—H312	0.9500
C3—C31	1.5109 (16)	C313—C314	1.3914 (19)
C4—C4A	1.4309 (17)	C313—C317	1.5082 (18)
C4—H4	0.9500	C314—C315	1.3931 (19)
C4A—C8A	1.3964 (17)	C314—H314	0.9500
C4A—C5	1.4053 (17)	C315—C316	1.3851 (18)
C5—C6	1.3841 (18)	C315—H315	0.9500
C5—H5	0.9500	C316—H316	0.9500
C6—C7	1.4074 (18)	C317—H31A	0.9800
C6—C61	1.5029 (17)	C317—H31B	0.9800
C7—C8	1.3810 (18)	C317—H31C	0.9800
C7—H7	0.9500		
			
C2—O1—C8A	122.61 (10)	O31—C31—C3	119.49 (11)
C31—N32—C311	128.30 (11)	N32—C31—C3	115.55 (11)
C31—N32—H32	115.8 (11)	C6—C61—H61A	109.5
C311—N32—H32	115.8 (12)	C6—C61—H61B	109.5
O2—C2—O1	116.01 (11)	H61A—C61—H61B	109.5
O2—C2—C3	126.69 (11)	C6—C61—H61C	109.5
O1—C2—C3	117.29 (11)	H61A—C61—H61C	109.5
C4—C3—C2	119.88 (11)	H61B—C61—H61C	109.5
C4—C3—C31	117.51 (11)	C312—C311—C316	120.00 (12)
C2—C3—C31	122.61 (11)	C312—C311—N32	123.55 (12)
C3—C4—C4A	121.71 (12)	C316—C311—N32	116.44 (11)
C3—C4—H4	119.1	C311—C312—C313	120.18 (12)
C4A—C4—H4	119.1	C311—C312—H312	119.9
C8A—C4A—C5	118.53 (11)	C313—C312—H312	119.9
C8A—C4A—C4	117.80 (12)	C314—C313—C312	119.43 (12)
C5—C4A—C4	123.68 (11)	C314—C313—C317	121.11 (12)
C6—C5—C4A	121.09 (12)	C312—C313—C317	119.41 (12)
C6—C5—H5	119.5	C313—C314—C315	120.21 (12)
C4A—C5—H5	119.5	C313—C314—H314	119.9
C5—C6—C7	118.26 (11)	C315—C314—H314	119.9
C5—C6—C61	121.07 (12)	C316—C315—C314	120.59 (13)
C7—C6—C61	120.66 (11)	C316—C315—H315	119.7
C8—C7—C6	122.08 (12)	C314—C315—H315	119.7
C8—C7—H7	119.0	C315—C316—C311	119.55 (12)
C6—C7—H7	119.0	C315—C316—H316	120.2
C7—C8—C8A	118.34 (12)	C311—C316—H316	120.2
C7—C8—H8	120.8	C313—C317—H31A	109.5
C8A—C8—H8	120.8	C313—C317—H31B	109.5
O1—C8A—C8	117.61 (11)	H31A—C317—H31B	109.5
O1—C8A—C4A	120.71 (11)	C313—C317—H31C	109.5
C8—C8A—C4A	121.68 (12)	H31A—C317—H31C	109.5
O31—C31—N32	124.96 (12)	H31B—C317—H31C	109.5
			
C8A—O1—C2—O2	179.71 (11)	C4—C4A—C8A—O1	0.84 (17)
C8A—O1—C2—C3	−0.18 (17)	C5—C4A—C8A—C8	0.31 (18)
O2—C2—C3—C4	−179.80 (12)	C4—C4A—C8A—C8	179.93 (11)
O1—C2—C3—C4	0.07 (18)	C311—N32—C31—O31	2.3 (2)
O2—C2—C3—C31	−0.5 (2)	C311—N32—C31—C3	−177.45 (11)
O1—C2—C3—C31	179.32 (10)	C4—C3—C31—O31	−4.22 (18)
C2—C3—C4—C4A	0.50 (19)	C2—C3—C31—O31	176.51 (12)
C31—C3—C4—C4A	−178.79 (11)	C4—C3—C31—N32	175.55 (11)
C3—C4—C4A—C8A	−0.95 (18)	C2—C3—C31—N32	−3.72 (17)
C3—C4—C4A—C5	178.65 (12)	C31—N32—C311—C312	−1.5 (2)
C8A—C4A—C5—C6	0.54 (18)	C31—N32—C311—C316	178.94 (12)
C4—C4A—C5—C6	−179.05 (11)	C316—C311—C312—C313	1.97 (19)
C4A—C5—C6—C7	−0.72 (18)	N32—C311—C312—C313	−177.58 (12)
C4A—C5—C6—C61	−179.92 (11)	C311—C312—C313—C314	−0.86 (19)
C5—C6—C7—C8	0.06 (19)	C311—C312—C313—C317	176.73 (12)
C61—C6—C7—C8	179.26 (12)	C312—C313—C314—C315	−0.84 (19)
C6—C7—C8—C8A	0.75 (19)	C317—C313—C314—C315	−178.39 (12)
C2—O1—C8A—C8	−179.42 (11)	C313—C314—C315—C316	1.4 (2)
C2—O1—C8A—C4A	−0.29 (18)	C314—C315—C316—C311	−0.3 (2)
C7—C8—C8A—O1	178.18 (11)	C312—C311—C316—C315	−1.37 (19)
C7—C8—C8A—C4A	−0.94 (19)	N32—C311—C316—C315	178.21 (11)
C5—C4A—C8A—O1	−178.78 (11)		

### (1) 6-Methyl-N-(3-methylphenyl)-2-oxo-2H-chromene-3-carboxamide Hydrogen-bond geometry (Å, º)

**Table:**

*D*—H···*A*	*D*—H	H···*A*	*D*···*A*	*D*—H···*A*
N32—H32···O2	0.893 (18)	1.957 (18)	2.7149 (14)	141.7 (16)
C312—H312···O31	0.95	2.26	2.8838 (16)	122
C5—H5···O1^i^	0.95	2.98	3.7304 (15)	137

Symmetry code: (i) *x*−1, *y*, *z*.

### (2) N-(3-Methoxyphenyl)-6-methyl-2-oxo-2H-chromene-3-carboxamide Crystal data

**Table:**

C_18_H_15_NO_4_	*Z* = 2
*M**_r_* = 309.31	*F*(000) = 324
Triclinic, *P*1	*D*_x_ = 1.439 Mg m^−^^3^
*a* = 7.1028 (4) Å	Mo *K*α radiation, λ = 0.71075 Å
*b* = 10.1367 (4) Å	Cell parameters from 9156 reflections
*c* = 10.8171 (5) Å	θ = 2.0–27.5°
α = 75.827 (4)°	µ = 0.10 mm^−^^1^
β = 88.318 (4)°	*T* = 100 K
γ = 71.271 (4)°	Needle, colourless
*V* = 714.10 (6) Å^3^	0.20 × 0.04 × 0.02 mm

### (2) N-(3-Methoxyphenyl)-6-methyl-2-oxo-2H-chromene-3-carboxamide Data collection

**Table:**

Rigaku AFC12 (Right) diffractometer	3262 independent reflections
Radiation source: Rotating Anode	2704 reflections with *I* > 2σ(*I*)
Confocal mirrors, HF Varimax monochromator	*R*_int_ = 0.025
Detector resolution: 28.5714 pixels mm^-1^	θ_max_ = 27.5°, θ_min_ = 2.0°
profile data from ω–scans	*h* = −9→9
Absorption correction: multi-scan (CrysAlis PRO; Rigaku Oxford Diffraction, 2015)	*k* = −13→13
*T*_min_ = 0.893, *T*_max_ = 1.000	*l* = −14→14
15638 measured reflections	

### (2) N-(3-Methoxyphenyl)-6-methyl-2-oxo-2H-chromene-3-carboxamide Refinement

**Table:**

Refinement on *F*^2^	0 restraints
Least-squares matrix: full	Hydrogen site location: mixed
*R*[*F*^2^ > 2σ(*F*^2^)] = 0.047	H atoms treated by a mixture of independent and constrained refinement
*wR*(*F*^2^) = 0.139	*w* = 1/[σ^2^(*F*_o_^2^) + (0.0823*P*)^2^ + 0.2203*P*] where *P* = (*F*_o_^2^ + 2*F*_c_^2^)/3
*S* = 1.02	(Δ/σ)_max_ = 0.001
3261 reflections	Δρ_max_ = 0.37 e Å^−^^3^
214 parameters	Δρ_min_ = −0.21 e Å^−^^3^

### (2) N-(3-Methoxyphenyl)-6-methyl-2-oxo-2H-chromene-3-carboxamide Special details

**Table:**

Experimental. CrysAlisPro 1.171.38.41 (Rigaku Oxford Diffraction, 2015) Empirical absorption correction using spherical harmonics, implemented in SCALE3 ABSPACK scaling algorithm.
Geometry. All esds (except the esd in the dihedral angle between two l.s. planes) are estimated using the full covariance matrix. The cell esds are taken into account individually in the estimation of esds in distances, angles and torsion angles; correlations between esds in cell parameters are only used when they are defined by crystal symmetry. An approximate (isotropic) treatment of cell esds is used for estimating esds involving l.s. planes.

### (2) N-(3-Methoxyphenyl)-6-methyl-2-oxo-2H-chromene-3-carboxamide Fractional atomic coordinates and isotropic or equivalent isotropic displacement parameters (Å^2^)

**Table:**

	*x*	*y*	*z*	*U*_iso_*/*U*_eq_	
O1	0.65788 (15)	0.44209 (11)	0.15778 (9)	0.0286 (3)	
O2	0.62079 (16)	0.61257 (11)	0.25621 (10)	0.0336 (3)	
O31	0.86393 (17)	0.30291 (11)	0.59953 (9)	0.0342 (3)	
N32	0.73898 (17)	0.54313 (13)	0.50493 (12)	0.0254 (3)	
H32	0.687 (3)	0.604 (2)	0.423 (2)	0.050 (6)*	
O313	0.68878 (17)	0.96846 (12)	0.62447 (11)	0.0367 (3)	
C8A	0.6993 (2)	0.30000 (15)	0.15628 (13)	0.0245 (3)	
C2	0.6741 (2)	0.48376 (15)	0.26677 (14)	0.0263 (3)	
C3	0.75233 (19)	0.37076 (14)	0.38259 (12)	0.0227 (3)	
C4	0.79282 (19)	0.23155 (15)	0.38073 (13)	0.0241 (3)	
H4	0.8409	0.1587	0.4573	0.029*	
C4A	0.76523 (19)	0.19084 (15)	0.26675 (13)	0.0232 (3)	
C5	0.8052 (2)	0.04818 (15)	0.25954 (13)	0.0249 (3)	
H5	0.8508	−0.0279	0.3343	0.030*	
C6	0.7792 (2)	0.01685 (15)	0.14495 (14)	0.0258 (3)	
C7	0.7102 (2)	0.13159 (16)	0.03699 (14)	0.0281 (3)	
H7	0.6904	0.1114	−0.0420	0.034*	
C8	0.6702 (2)	0.27245 (16)	0.04094 (14)	0.0293 (3)	
H8	0.6238	0.3486	−0.0336	0.035*	
C31	0.7903 (2)	0.40253 (15)	0.50651 (13)	0.0250 (3)	
C61	0.8246 (2)	−0.13560 (16)	0.13511 (15)	0.0325 (3)	
H61A	0.7066	−0.1464	0.0995	0.049*	
H61B	0.8615	−0.2012	0.2202	0.049*	
H61C	0.9351	−0.1586	0.0792	0.049*	
C311	0.75735 (19)	0.60513 (16)	0.60585 (13)	0.0252 (3)	
C312	0.7157 (2)	0.75307 (16)	0.57413 (14)	0.0268 (3)	
H312	0.6788	0.8065	0.4882	0.032*	
C313	0.7276 (2)	0.82377 (16)	0.66755 (14)	0.0289 (3)	
C314	0.7787 (2)	0.74642 (17)	0.79326 (14)	0.0321 (3)	
H314	0.7861	0.7937	0.8578	0.039*	
C315	0.8186 (2)	0.59968 (18)	0.82285 (15)	0.0356 (4)	
H315	0.8534	0.5465	0.9090	0.043*	
C316	0.8099 (2)	0.52680 (17)	0.73131 (14)	0.0318 (3)	
H316	0.8392	0.4254	0.7540	0.038*	
C317	0.6726 (2)	1.04748 (18)	0.71900 (16)	0.0368 (4)	
H31A	0.6357	1.1504	0.6774	0.055*	
H31B	0.8008	1.0162	0.7670	0.055*	
H31C	0.5703	1.0302	0.7775	0.055*	

### (2) N-(3-Methoxyphenyl)-6-methyl-2-oxo-2H-chromene-3-carboxamide Atomic displacement parameters (Å^2^)

**Table:**

	*U*^11^	*U*^22^	*U*^33^	*U*^12^	*U*^13^	*U*^23^
O1	0.0367 (6)	0.0231 (5)	0.0253 (5)	−0.0075 (4)	−0.0064 (4)	−0.0066 (4)
O2	0.0443 (6)	0.0214 (5)	0.0336 (6)	−0.0075 (4)	−0.0094 (5)	−0.0070 (4)
O31	0.0488 (7)	0.0300 (6)	0.0241 (5)	−0.0119 (5)	−0.0046 (4)	−0.0073 (4)
N32	0.0263 (6)	0.0262 (6)	0.0261 (6)	−0.0088 (5)	0.0001 (5)	−0.0103 (5)
O313	0.0481 (7)	0.0317 (6)	0.0376 (6)	−0.0158 (5)	0.0010 (5)	−0.0181 (5)
C8A	0.0228 (6)	0.0233 (7)	0.0285 (7)	−0.0071 (5)	−0.0009 (5)	−0.0083 (5)
C2	0.0260 (7)	0.0274 (7)	0.0274 (7)	−0.0093 (6)	−0.0027 (5)	−0.0092 (6)
C3	0.0203 (6)	0.0251 (7)	0.0242 (7)	−0.0083 (5)	0.0010 (5)	−0.0073 (5)
C4	0.0222 (6)	0.0266 (7)	0.0238 (7)	−0.0082 (5)	0.0005 (5)	−0.0066 (5)
C4A	0.0194 (6)	0.0280 (7)	0.0248 (7)	−0.0091 (5)	0.0023 (5)	−0.0099 (5)
C5	0.0245 (7)	0.0250 (7)	0.0261 (7)	−0.0086 (5)	0.0025 (5)	−0.0072 (5)
C6	0.0226 (6)	0.0277 (7)	0.0314 (7)	−0.0098 (5)	0.0047 (5)	−0.0132 (6)
C7	0.0276 (7)	0.0338 (8)	0.0256 (7)	−0.0098 (6)	0.0002 (5)	−0.0122 (6)
C8	0.0320 (7)	0.0295 (7)	0.0251 (7)	−0.0077 (6)	−0.0033 (6)	−0.0071 (6)
C31	0.0241 (7)	0.0278 (7)	0.0255 (7)	−0.0096 (5)	0.0016 (5)	−0.0094 (5)
C61	0.0359 (8)	0.0300 (8)	0.0363 (8)	−0.0114 (6)	0.0040 (6)	−0.0156 (6)
C311	0.0199 (6)	0.0322 (7)	0.0293 (7)	−0.0106 (5)	0.0035 (5)	−0.0154 (6)
C312	0.0247 (7)	0.0308 (7)	0.0284 (7)	−0.0101 (6)	0.0012 (5)	−0.0119 (6)
C313	0.0241 (7)	0.0323 (8)	0.0367 (8)	−0.0126 (6)	0.0051 (6)	−0.0161 (6)
C314	0.0304 (8)	0.0440 (9)	0.0309 (8)	−0.0156 (7)	0.0060 (6)	−0.0211 (7)
C315	0.0390 (8)	0.0434 (9)	0.0264 (7)	−0.0136 (7)	0.0031 (6)	−0.0118 (6)
C316	0.0339 (8)	0.0339 (8)	0.0293 (8)	−0.0113 (6)	0.0025 (6)	−0.0109 (6)
C317	0.0360 (8)	0.0392 (9)	0.0471 (9)	−0.0166 (7)	0.0072 (7)	−0.0273 (7)

### (2) N-(3-Methoxyphenyl)-6-methyl-2-oxo-2H-chromene-3-carboxamide Geometric parameters (Å, º)

**Table:**

O1—C2	1.3656 (16)	C6—C61	1.5040 (19)
O1—C8A	1.3785 (16)	C7—C8	1.375 (2)
O2—C2	1.2137 (17)	C7—H7	0.9500
O31—C31	1.2247 (17)	C8—H8	0.9500
N32—C31	1.3488 (18)	C61—H61A	0.9800
N32—C311	1.4145 (17)	C61—H61B	0.9800
N32—H32	0.96 (2)	C61—H61C	0.9800
O313—C313	1.3629 (18)	C311—C312	1.387 (2)
O313—C317	1.4267 (17)	C311—C316	1.387 (2)
C8A—C8	1.3785 (19)	C312—C313	1.3931 (19)
C8A—C4A	1.3871 (19)	C312—H312	0.9500
C2—C3	1.4560 (19)	C313—C314	1.386 (2)
C3—C4	1.3518 (19)	C314—C315	1.377 (2)
C3—C31	1.5038 (18)	C314—H314	0.9500
C4—C4A	1.4297 (18)	C315—C316	1.386 (2)
C4—H4	0.9500	C315—H315	0.9500
C4A—C5	1.4028 (19)	C316—H316	0.9500
C5—C6	1.3841 (19)	C317—H31A	0.9800
C5—H5	0.9500	C317—H31B	0.9800
C6—C7	1.400 (2)	C317—H31C	0.9800
			
C2—O1—C8A	122.60 (11)	O31—C31—C3	119.53 (12)
C31—N32—C311	128.29 (13)	N32—C31—C3	115.57 (12)
C31—N32—H32	112.4 (12)	C6—C61—H61A	109.5
C311—N32—H32	119.3 (12)	C6—C61—H61B	109.5
C313—O313—C317	116.71 (12)	H61A—C61—H61B	109.5
O1—C8A—C8	116.95 (12)	C6—C61—H61C	109.5
O1—C8A—C4A	120.97 (12)	H61A—C61—H61C	109.5
C8—C8A—C4A	122.07 (13)	H61B—C61—H61C	109.5
O2—C2—O1	115.82 (12)	C312—C311—C316	120.02 (13)
O2—C2—C3	126.86 (13)	C312—C311—N32	116.32 (13)
O1—C2—C3	117.32 (12)	C316—C311—N32	123.65 (14)
C4—C3—C2	119.74 (12)	C311—C312—C313	120.38 (14)
C4—C3—C31	117.86 (12)	C311—C312—H312	119.8
C2—C3—C31	122.40 (12)	C313—C312—H312	119.8
C3—C4—C4A	121.83 (13)	O313—C313—C314	124.95 (13)
C3—C4—H4	119.1	O313—C313—C312	115.09 (13)
C4A—C4—H4	119.1	C314—C313—C312	119.96 (14)
C8A—C4A—C5	118.50 (12)	C315—C314—C313	118.75 (13)
C8A—C4A—C4	117.36 (12)	C315—C314—H314	120.6
C5—C4A—C4	124.12 (13)	C313—C314—H314	120.6
C6—C5—C4A	120.84 (13)	C314—C315—C316	122.32 (15)
C6—C5—H5	119.6	C314—C315—H315	118.8
C4A—C5—H5	119.6	C316—C315—H315	118.8
C5—C6—C7	118.07 (13)	C315—C316—C311	118.57 (15)
C5—C6—C61	121.52 (13)	C315—C316—H316	120.7
C7—C6—C61	120.41 (13)	C311—C316—H316	120.7
C8—C7—C6	122.46 (13)	O313—C317—H31A	109.5
C8—C7—H7	118.8	O313—C317—H31B	109.5
C6—C7—H7	118.8	H31A—C317—H31B	109.5
C7—C8—C8A	118.06 (13)	O313—C317—H31C	109.5
C7—C8—H8	121.0	H31A—C317—H31C	109.5
C8A—C8—H8	121.0	H31B—C317—H31C	109.5
O31—C31—N32	124.90 (13)		
			
C2—O1—C8A—C8	177.70 (12)	O1—C8A—C8—C7	−179.94 (12)
C2—O1—C8A—C4A	−1.7 (2)	C4A—C8A—C8—C7	−0.6 (2)
C8A—O1—C2—O2	−175.39 (12)	C311—N32—C31—O31	−0.1 (2)
C8A—O1—C2—C3	4.54 (19)	C311—N32—C31—C3	−179.84 (12)
O2—C2—C3—C4	175.53 (13)	C4—C3—C31—O31	3.1 (2)
O1—C2—C3—C4	−4.39 (19)	C2—C3—C31—O31	−177.02 (13)
O2—C2—C3—C31	−4.3 (2)	C4—C3—C31—N32	−177.06 (11)
O1—C2—C3—C31	175.77 (11)	C2—C3—C31—N32	2.78 (19)
C2—C3—C4—C4A	1.4 (2)	C31—N32—C311—C312	172.29 (12)
C31—C3—C4—C4A	−178.73 (11)	C31—N32—C311—C316	−9.0 (2)
O1—C8A—C4A—C5	−179.94 (11)	C316—C311—C312—C313	0.5 (2)
C8—C8A—C4A—C5	0.7 (2)	N32—C311—C312—C313	179.28 (12)
O1—C8A—C4A—C4	−1.47 (19)	C317—O313—C313—C314	−9.2 (2)
C8—C8A—C4A—C4	179.19 (12)	C317—O313—C313—C312	171.67 (12)
C3—C4—C4A—C8A	1.5 (2)	C311—C312—C313—O313	178.26 (12)
C3—C4—C4A—C5	179.88 (12)	C311—C312—C313—C314	−0.9 (2)
C8A—C4A—C5—C6	−0.1 (2)	O313—C313—C314—C315	−178.48 (13)
C4—C4A—C5—C6	−178.51 (12)	C312—C313—C314—C315	0.6 (2)
C4A—C5—C6—C7	−0.5 (2)	C313—C314—C315—C316	0.1 (2)
C4A—C5—C6—C61	178.98 (12)	C314—C315—C316—C311	−0.6 (2)
C5—C6—C7—C8	0.7 (2)	C312—C311—C316—C315	0.2 (2)
C61—C6—C7—C8	−178.82 (13)	N32—C311—C316—C315	−178.48 (13)
C6—C7—C8—C8A	−0.1 (2)		

### (2) N-(3-Methoxyphenyl)-6-methyl-2-oxo-2H-chromene-3-carboxamide Hydrogen-bond geometry (Å, º)

**Table:**

*D*—H···*A*	*D*—H	H···*A*	*D*···*A*	*D*—H···*A*
N32—H32···O2	0.96 (2)	1.85 (2)	2.6952 (16)	145.7 (17)
C8—H8···O1^i^	0.95	2.52	3.3676 (18)	149
C61—H61*B*···O31^ii^	0.98	2.57	3.4044 (19)	143
C317—H31*A*···O31^iii^	0.98	2.57	3.2769 (19)	129

Symmetry codes: (i) −*x*+1, −*y*+1, −*z*; (ii) −*x*+2, −*y*, −*z*+1; (iii) *x*, *y*+1, *z*.

### (3) 6-Methoxy-N-(3-methoxyphenyl)-2-oxo-2H-chromene-3-carboxamide Crystal data

**Table:**

C_18_H_15_NO_5_	*Z* = 2
*M**_r_* = 325.31	*F*(000) = 340
Triclinic, *P*1	*D*_x_ = 1.483 Mg m^−^^3^
*a* = 6.7722 (5) Å	Mo *K*α radiation, λ = 0.71073 Å
*b* = 8.3098 (7) Å	Cell parameters from 3630 reflections
*c* = 14.4202 (13) Å	θ = 2.7–27.4°
α = 91.874 (7)°	µ = 0.11 mm^−^^1^
β = 100.009 (7)°	*T* = 100 K
γ = 113.042 (7)°	Plate, yellow
*V* = 730.84 (11) Å^3^	0.17 × 0.11 × 0.02 mm

### (3) 6-Methoxy-N-(3-methoxyphenyl)-2-oxo-2H-chromene-3-carboxamide Data collection

**Table:**

Rigaku AFC12 (Right) diffractometer	3302 independent reflections
Radiation source: Rotating Anode, Rotating Anode	2666 reflections with *I* > 2σ(*I*)
Confocal mirrors, HF Varimax monochromator	*R*_int_ = 0.033
Detector resolution: 28.5714 pixels mm^-1^	θ_max_ = 27.5°, θ_min_ = 2.7°
profile data from ω–scans	*h* = −7→8
Absorption correction: multi-scan (CrysAlis PRO; Rigaku Oxford Diffraction, 2015)	*k* = −10→9
*T*_min_ = 0.792, *T*_max_ = 1.000	*l* = −18→18
8745 measured reflections	

### (3) 6-Methoxy-N-(3-methoxyphenyl)-2-oxo-2H-chromene-3-carboxamide Refinement

**Table:**

Refinement on *F*^2^	0 restraints
Least-squares matrix: full	Hydrogen site location: mixed
*R*[*F*^2^ > 2σ(*F*^2^)] = 0.071	H atoms treated by a mixture of independent and constrained refinement
*wR*(*F*^2^) = 0.152	*w* = 1/[σ^2^(*F*_o_^2^) + (0.0606*P*)^2^ + 0.3539*P*] where *P* = (*F*_o_^2^ + 2*F*_c_^2^)/3
*S* = 1.16	(Δ/σ)_max_ < 0.001
3302 reflections	Δρ_max_ = 0.25 e Å^−^^3^
223 parameters	Δρ_min_ = −0.26 e Å^−^^3^

### (3) 6-Methoxy-N-(3-methoxyphenyl)-2-oxo-2H-chromene-3-carboxamide Special details

**Table:**

Experimental. CrysAlisPro 1.171.38.41 (Rigaku Oxford Diffraction, 2015) Empirical absorption correction using spherical harmonics, implemented in SCALE3 ABSPACK scaling algorithm.
Geometry. All esds (except the esd in the dihedral angle between two l.s. planes) are estimated using the full covariance matrix. The cell esds are taken into account individually in the estimation of esds in distances, angles and torsion angles; correlations between esds in cell parameters are only used when they are defined by crystal symmetry. An approximate (isotropic) treatment of cell esds is used for estimating esds involving l.s. planes.

### (3) 6-Methoxy-N-(3-methoxyphenyl)-2-oxo-2H-chromene-3-carboxamide Fractional atomic coordinates and isotropic or equivalent isotropic displacement parameters (Å^2^)

**Table:**

	*x*	*y*	*z*	*U*_iso_*/*U*_eq_	
O1	0.7758 (2)	0.24765 (19)	0.52564 (10)	0.0207 (3)	
O2	0.9491 (2)	0.3631 (2)	0.41317 (11)	0.0227 (4)	
O6	0.0500 (2)	0.0132 (2)	0.68826 (11)	0.0244 (4)	
O31	0.4048 (2)	0.4552 (2)	0.27644 (11)	0.0249 (4)	
O313	0.5339 (3)	0.7656 (2)	0.01169 (11)	0.0272 (4)	
N32	0.7594 (3)	0.4985 (2)	0.27488 (13)	0.0212 (4)	
H32	0.872 (4)	0.476 (3)	0.3080 (18)	0.032 (7)*	
C2	0.7823 (3)	0.3270 (3)	0.44407 (15)	0.0195 (5)	
C3	0.5890 (3)	0.3567 (3)	0.40191 (15)	0.0183 (4)	
C4	0.4121 (3)	0.3038 (3)	0.44288 (15)	0.0193 (5)	
H4	0.2872	0.3236	0.4147	0.023*	
C4A	0.4085 (3)	0.2184 (3)	0.52792 (15)	0.0188 (5)	
C5	0.2277 (3)	0.1573 (3)	0.57142 (16)	0.0200 (5)	
H5	0.0987	0.1733	0.5454	0.024*	
C6	0.2368 (3)	0.0732 (3)	0.65245 (15)	0.0203 (5)	
C7	0.4289 (4)	0.0548 (3)	0.69251 (16)	0.0214 (5)	
H7	0.4362	0.0012	0.7495	0.026*	
C8	0.6098 (4)	0.1144 (3)	0.64972 (15)	0.0213 (5)	
H8	0.7399	0.1004	0.6763	0.026*	
C8A	0.5963 (3)	0.1941 (3)	0.56802 (15)	0.0190 (5)	
C31	0.5759 (3)	0.4431 (3)	0.31191 (15)	0.0203 (5)	
C61	0.0465 (4)	−0.0852 (3)	0.76813 (16)	0.0252 (5)	
H61A	−0.0977	−0.1238	0.7851	0.038*	
H61B	0.1593	−0.0108	0.8219	0.038*	
H61C	0.0755	−0.1882	0.7520	0.038*	
C311	0.7911 (3)	0.5766 (3)	0.19019 (15)	0.0203 (5)	
C312	0.6436 (3)	0.6337 (3)	0.13821 (15)	0.0210 (5)	
H312	0.5130	0.6212	0.1588	0.025*	
C313	0.6884 (3)	0.7093 (3)	0.05573 (16)	0.0216 (5)	
C314	0.8761 (4)	0.7263 (3)	0.02325 (16)	0.0239 (5)	
H314	0.9040	0.7762	−0.0339	0.029*	
C315	1.0229 (4)	0.6682 (3)	0.07684 (17)	0.0256 (5)	
H315	1.1530	0.6802	0.0559	0.031*	
C316	0.9831 (4)	0.5942 (3)	0.15912 (16)	0.0239 (5)	
H316	1.0846	0.5553	0.1947	0.029*	
C317	0.5672 (4)	0.8430 (3)	−0.07431 (17)	0.0296 (5)	
H31A	0.4426	0.8715	−0.1002	0.044*	
H31B	0.5798	0.7598	−0.1203	0.044*	
H31C	0.7020	0.9508	−0.0615	0.044*	

### (3) 6-Methoxy-N-(3-methoxyphenyl)-2-oxo-2H-chromene-3-carboxamide Atomic displacement parameters (Å^2^)

**Table:**

	*U*^11^	*U*^22^	*U*^33^	*U*^12^	*U*^13^	*U*^23^
O1	0.0169 (8)	0.0216 (8)	0.0281 (8)	0.0112 (6)	0.0064 (6)	0.0086 (6)
O2	0.0189 (8)	0.0229 (8)	0.0302 (9)	0.0109 (7)	0.0078 (6)	0.0075 (6)
O6	0.0193 (8)	0.0271 (9)	0.0313 (9)	0.0115 (7)	0.0101 (6)	0.0116 (7)
O31	0.0169 (8)	0.0281 (9)	0.0315 (9)	0.0101 (7)	0.0058 (6)	0.0106 (7)
O313	0.0262 (9)	0.0306 (9)	0.0318 (9)	0.0168 (7)	0.0091 (7)	0.0130 (7)
N32	0.0176 (10)	0.0238 (10)	0.0267 (10)	0.0116 (8)	0.0068 (8)	0.0081 (8)
C2	0.0186 (11)	0.0131 (10)	0.0254 (11)	0.0051 (8)	0.0044 (8)	0.0014 (8)
C3	0.0172 (10)	0.0133 (10)	0.0252 (11)	0.0069 (8)	0.0047 (8)	0.0025 (8)
C4	0.0182 (11)	0.0133 (10)	0.0268 (12)	0.0076 (8)	0.0022 (8)	0.0027 (8)
C4A	0.0199 (11)	0.0126 (10)	0.0250 (11)	0.0078 (8)	0.0048 (8)	0.0010 (8)
C5	0.0153 (10)	0.0172 (11)	0.0288 (12)	0.0088 (8)	0.0023 (8)	0.0016 (9)
C6	0.0185 (11)	0.0162 (11)	0.0267 (12)	0.0068 (9)	0.0063 (9)	0.0021 (8)
C7	0.0230 (12)	0.0187 (11)	0.0243 (11)	0.0091 (9)	0.0068 (9)	0.0052 (9)
C8	0.0185 (11)	0.0175 (11)	0.0294 (12)	0.0095 (9)	0.0026 (9)	0.0041 (9)
C8A	0.0157 (10)	0.0143 (10)	0.0274 (12)	0.0054 (8)	0.0069 (8)	0.0020 (8)
C31	0.0181 (11)	0.0160 (11)	0.0267 (12)	0.0072 (9)	0.0035 (9)	0.0022 (9)
C61	0.0258 (12)	0.0226 (12)	0.0290 (12)	0.0092 (10)	0.0106 (9)	0.0083 (9)
C311	0.0206 (11)	0.0135 (10)	0.0254 (11)	0.0051 (9)	0.0055 (8)	0.0014 (8)
C312	0.0190 (11)	0.0167 (11)	0.0293 (12)	0.0077 (9)	0.0084 (9)	0.0049 (9)
C313	0.0190 (11)	0.0171 (11)	0.0283 (12)	0.0073 (9)	0.0036 (9)	0.0018 (9)
C314	0.0256 (12)	0.0210 (12)	0.0262 (12)	0.0088 (9)	0.0088 (9)	0.0070 (9)
C315	0.0182 (11)	0.0252 (12)	0.0346 (13)	0.0076 (9)	0.0110 (9)	0.0060 (10)
C316	0.0201 (11)	0.0216 (12)	0.0308 (13)	0.0099 (9)	0.0036 (9)	0.0044 (9)
C317	0.0308 (13)	0.0296 (13)	0.0301 (13)	0.0129 (11)	0.0069 (10)	0.0129 (10)

### (3) 6-Methoxy-N-(3-methoxyphenyl)-2-oxo-2H-chromene-3-carboxamide Geometric parameters (Å, º)

**Table:**

O1—C2	1.366 (2)	C7—C8	1.391 (3)
O1—C8A	1.379 (2)	C7—H7	0.9500
O2—C2	1.218 (2)	C8—C8A	1.377 (3)
O6—C6	1.366 (3)	C8—H8	0.9500
O6—C61	1.432 (3)	C61—H61A	0.9800
O31—C31	1.226 (3)	C61—H61B	0.9800
O313—C313	1.374 (3)	C61—H61C	0.9800
O313—C317	1.428 (3)	C311—C312	1.385 (3)
N32—C31	1.356 (3)	C311—C316	1.404 (3)
N32—C311	1.412 (3)	C312—C313	1.388 (3)
N32—H32	0.92 (3)	C312—H312	0.9500
C2—C3	1.459 (3)	C313—C314	1.388 (3)
C3—C4	1.352 (3)	C314—C315	1.397 (3)
C3—C31	1.509 (3)	C314—H314	0.9500
C4—C4A	1.436 (3)	C315—C316	1.374 (3)
C4—H4	0.9500	C315—H315	0.9500
C4A—C8A	1.394 (3)	C316—H316	0.9500
C4A—C5	1.397 (3)	C317—H31A	0.9800
C5—C6	1.385 (3)	C317—H31B	0.9800
C5—H5	0.9500	C317—H31C	0.9800
C6—C7	1.397 (3)		
			
C2—O1—C8A	123.06 (16)	O31—C31—N32	124.7 (2)
C6—O6—C61	117.74 (17)	O31—C31—C3	119.63 (19)
C313—O313—C317	117.52 (18)	N32—C31—C3	115.61 (18)
C31—N32—C311	127.96 (19)	O6—C61—H61A	109.5
C31—N32—H32	114.6 (16)	O6—C61—H61B	109.5
C311—N32—H32	117.4 (16)	H61A—C61—H61B	109.5
O2—C2—O1	116.03 (18)	O6—C61—H61C	109.5
O2—C2—C3	126.7 (2)	H61A—C61—H61C	109.5
O1—C2—C3	117.27 (18)	H61B—C61—H61C	109.5
C4—C3—C2	119.95 (19)	C312—C311—C316	120.1 (2)
C4—C3—C31	117.69 (18)	C312—C311—N32	123.3 (2)
C2—C3—C31	122.35 (18)	C316—C311—N32	116.59 (19)
C3—C4—C4A	121.62 (19)	C311—C312—C313	119.3 (2)
C3—C4—H4	119.2	C311—C312—H312	120.3
C4A—C4—H4	119.2	C313—C312—H312	120.3
C8A—C4A—C5	118.77 (19)	O313—C313—C314	124.3 (2)
C8A—C4A—C4	117.53 (19)	O313—C313—C312	114.19 (19)
C5—C4A—C4	123.69 (19)	C314—C313—C312	121.5 (2)
C6—C5—C4A	119.93 (19)	C313—C314—C315	118.3 (2)
C6—C5—H5	120.0	C313—C314—H314	120.9
C4A—C5—H5	120.0	C315—C314—H314	120.9
O6—C6—C5	115.76 (19)	C316—C315—C314	121.4 (2)
O6—C6—C7	124.16 (19)	C316—C315—H315	119.3
C5—C6—C7	120.1 (2)	C314—C315—H315	119.3
C8—C7—C6	120.5 (2)	C315—C316—C311	119.4 (2)
C8—C7—H7	119.7	C315—C316—H316	120.3
C6—C7—H7	119.7	C311—C316—H316	120.3
C8A—C8—C7	118.6 (2)	O313—C317—H31A	109.5
C8A—C8—H8	120.7	O313—C317—H31B	109.5
C7—C8—H8	120.7	H31A—C317—H31B	109.5
C8—C8A—O1	117.40 (18)	O313—C317—H31C	109.5
C8—C8A—C4A	122.04 (19)	H31A—C317—H31C	109.5
O1—C8A—C4A	120.55 (19)	H31B—C317—H31C	109.5
			
C8A—O1—C2—O2	−178.07 (17)	C4—C4A—C8A—C8	179.69 (19)
C8A—O1—C2—C3	0.7 (3)	C5—C4A—C8A—O1	178.01 (18)
O2—C2—C3—C4	177.7 (2)	C4—C4A—C8A—O1	−1.3 (3)
O1—C2—C3—C4	−1.0 (3)	C311—N32—C31—O31	−1.1 (4)
O2—C2—C3—C31	−1.4 (3)	C311—N32—C31—C3	177.28 (19)
O1—C2—C3—C31	−179.99 (18)	C4—C3—C31—O31	−3.4 (3)
C2—C3—C4—C4A	0.1 (3)	C2—C3—C31—O31	175.63 (19)
C31—C3—C4—C4A	179.18 (18)	C4—C3—C31—N32	178.15 (18)
C3—C4—C4A—C8A	1.0 (3)	C2—C3—C31—N32	−2.8 (3)
C3—C4—C4A—C5	−178.2 (2)	C31—N32—C311—C312	10.4 (3)
C8A—C4A—C5—C6	−0.5 (3)	C31—N32—C311—C316	−169.2 (2)
C4—C4A—C5—C6	178.8 (2)	C316—C311—C312—C313	−0.5 (3)
C61—O6—C6—C5	175.76 (18)	N32—C311—C312—C313	179.91 (19)
C61—O6—C6—C7	−4.4 (3)	C317—O313—C313—C314	1.5 (3)
C4A—C5—C6—O6	−178.04 (18)	C317—O313—C313—C312	−179.27 (19)
C4A—C5—C6—C7	2.2 (3)	C311—C312—C313—O313	−178.17 (19)
O6—C6—C7—C8	177.8 (2)	C311—C312—C313—C314	1.1 (3)
C5—C6—C7—C8	−2.4 (3)	O313—C313—C314—C315	178.0 (2)
C6—C7—C8—C8A	1.0 (3)	C312—C313—C314—C315	−1.2 (3)
C7—C8—C8A—O1	−178.30 (18)	C313—C314—C315—C316	0.7 (3)
C7—C8—C8A—C4A	0.8 (3)	C314—C315—C316—C311	−0.1 (3)
C2—O1—C8A—C8	179.51 (19)	C312—C311—C316—C315	0.0 (3)
C2—O1—C8A—C4A	0.4 (3)	N32—C311—C316—C315	179.6 (2)
C5—C4A—C8A—C8	−1.0 (3)		

### (3) 6-Methoxy-N-(3-methoxyphenyl)-2-oxo-2H-chromene-3-carboxamide Hydrogen-bond geometry (Å, º)

**Table:**

*D*—H···*A*	*D*—H	H···*A*	*D*···*A*	*D*—H···*A*
N32—H32···O2	0.92 (3)	1.91 (3)	2.699 (2)	143 (2)
C4—H4···O2^i^	0.95	2.43	3.319 (3)	155
C5—H5···O1^i^	0.95	2.47	3.391 (3)	164
C8—H8···O6^ii^	0.95	2.46	3.364 (3)	160
C312—H312···O31	0.95	2.26	2.868 (3)	121
C315—H315···O313^ii^	0.95	2.59	3.536 (4)	171

Symmetry codes: (i) *x*−1, *y*, *z*; (ii) *x*+1, *y*, *z*.
