# Supplementary material for: Crystal structures of three 6-substituted coumarin-3-carboxamide derivatives
Source: Acta Crystallogr E Crystallogr Commun. 2016 Jun 10;72(Pt 7):926–32. doi: 10.1107/S2056989016008665 (PMC4992908; doi:10.1107/S2056989016008665)

Search Overview

Search:	search3
Date/Time done:	Tue May 10 10:50:13 2016
Database(s):	CSD version 5.37 updates (Nov 2015)<br>CSD version 5.37 (November 2015)<br>CSD version 5.37 (November 2015)<br>CSD version 5.37 updates (Feb 2016)
Restriction Info:	No refcode restrictions applied
Filters:	3D coordinates determined<br>No errors<br>No ions<br>Only Organics<br>Not disordered<br>Not polymeric<br>No powder structures
Percentage Completed:	100%
Number of Hits:	35

Single query used. Search found structures that:

match

Query 2

Query 2

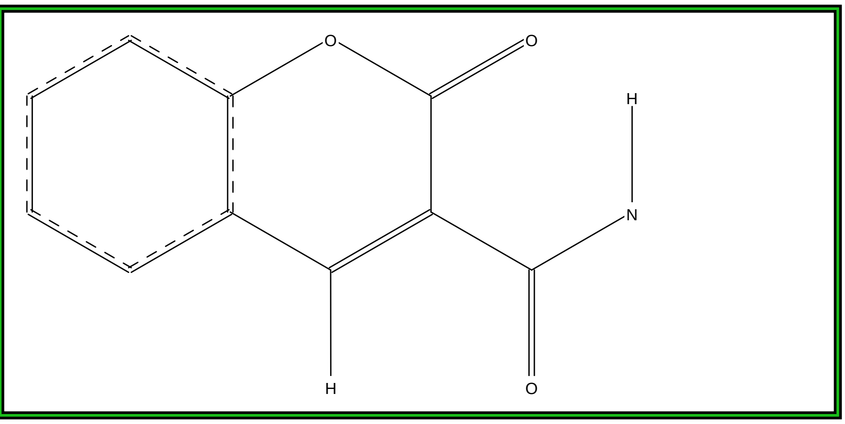

ACEMEB

Reference: E.V.Garcia-Baez, F.J.Martinez-Martinez, H.Hopfl, I.I.Padilla-Martinez (2003) *ARKIVOC* ,**4,**100-11

Formula: $C_7 H_6 N_2 S_1$,$C_{18} H_{15} N_1 O_3$,0.5($C_6 H_6$)

Compound Name: 2-Aminobenzothiazole N-((2-oxo-2H-1-benzopyran-3-yl)carboxyl) phenethylamide benzene solvate

Space Group:	P-1	***Cell:***	***a*** 6.690(0)	***b*** 12.730(0)	***c*** 14.164(0)
Space Group No.:	2	***(Å,°)***	α 91.07(0)	β 100.35(0)	γ 98.64(0)
R-Factor (%):	5.45	***Temperature(K):*** 100		***Density(g/cm³):*** 1.368	

Parameters

Fragment 1

**N3O2 (D)**	2.729
**NHO (A)**	135.949
**H3O2 (D)**	2.027
**N3H3 (D)**	0.880

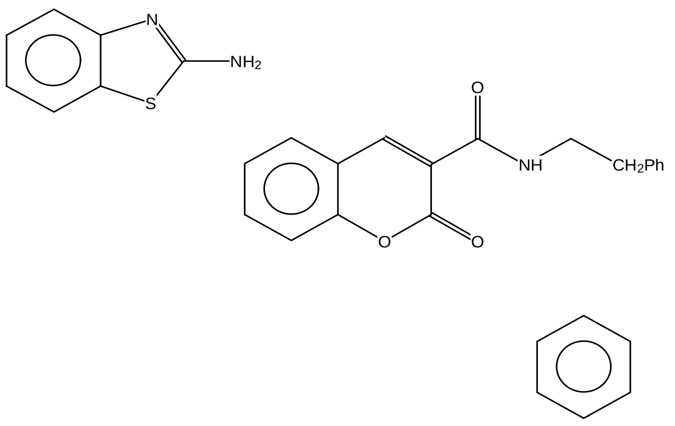

ADAJOF

Reference: Yan Zhan, Shu-Kun Lin (2006) *Acta Crystallogr.,Sect.E:Struct.Rep.Online* ,**62,**o2097

Formula: $C_{17} H_{12} N_2 O_5$

Compound Name: N'-(2-Hydroxybenzoyl)-2-oxo-2H-chromene-3-carbohydrazide

Space Group:	P21/c	***Cell:***	***a*** 9.467(4)	***b*** 11.766(4)	***c*** 13.268(7)
Space Group No.:	14	***(Å,°)***	α 90.00	β 100.96(2)	γ 90.00
R-Factor (%):	4.00	***Temperature(K):*** 293		***Density(g/cm³):*** 1.485	

Parameters

Fragment 1

**N3O2 (D)**	2.639
**NHO (A)**	134.371
**H3O2 (D)**	1.904
**N3H3 (D)**	0.930

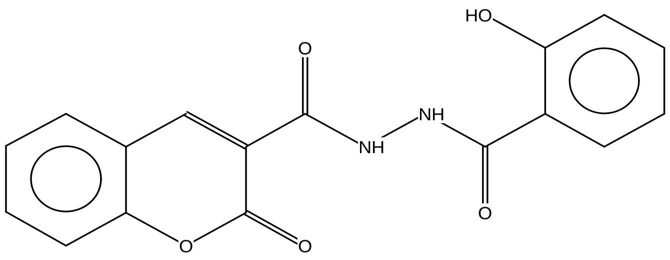

BONKAS

Reference:	O.Julien, M.Kampmann, M.C.Bassik, J.A.Zorn, V.J.Venditto, K.Shimbo, N.J.Agard, K.Shimada, A.L.Rheingold, B.R.Stockwell, J.S.Weissman, J.A.Wells (2014) *Nat.Chem.Biol.* ,**10,**969
Formula:	C_{24} H_{17} N_3 O_4
Compound Name:	N-(3-(Imidazo[1,2-a]pyridin-2-yl)phenyl)-8-methoxy-2-oxo-2H-chromene-3-carboxamide

Space Group:	C2/c	***Cell:***	***a*** 36.299(4)	***b*** 6.374(0)	***c*** 18.828(2)
Space Group No.:	15	***(Å,°)***	α 90.00	β 121.05(0)	γ 90.00
R-Factor (%):	3.18	***Temperature(K):*** 100		***Density(g/cm³):*** 1.464	

Parameters

Fragment 1	
**N3O2 (D)**	2.682
**NHO (A)**	143.556
**H3O2 (D)**	1.923
**N3H3 (D)**	0.879

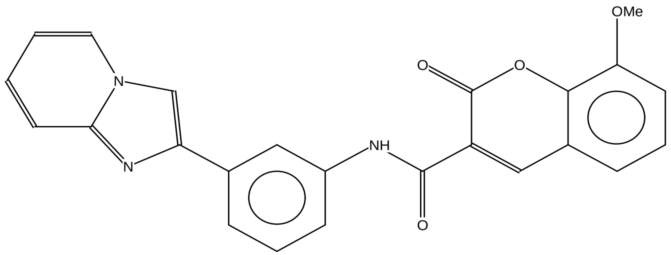

DISXUA

Reference:	M.Maldonado-Dominguez, R.Arcos-Ramos, M.Romero, B.Flores-Perez, N.Farfan, R.Santillan, P.G.Lacroix, I.Malfant (2014) *New J.Chem.* ,**38,**260
Formula:	C_{20} H_{19} F_1 N_2 O_3
Compound Name:	7-(Diethylamino)-N-(4-fluorophenyl)-2-oxo-2H-chromene-3-carboxamide

Space Group:	P-1	***Cell:***	***a*** 6.950(0)	***b*** 9.491(0)	***c*** 13.286(1)
Space Group No.:	2	***(Å,°)***	α 104.26(0)	β 91.62(0)	γ 100.78(0)
R-Factor (%):	4.93	***Temperature(K):*** 132		***Density(g/cm³):*** 1.415	

Parameters

Fragment 1	
**N3O2 (D)**	2.684
**NHO (A)**	145.762
**H3O2 (D)**	1.895
**N3H3 (D)**	0.896

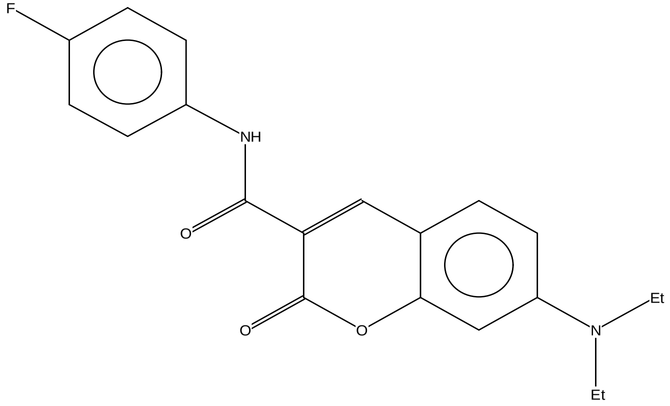

Search: search3 (Tue May 10 10:50:13 2016): Hits 5-6

DISYAH

Reference:	M.Maldonado-Dominguez, R.Arcos-Ramos, M.Romero, B.Flores-Perez, N.Farfan, R.Santillan, P.G.Lacroix, I.Malfant (2014) *New J.Chem.* ,**38,**260
Formula:	$C_{22} H_{22} N_2 O_5$
Compound Name:	Methyl 4-(((7-(diethylamino)-2-oxo-2H-chromen-3-yl)carbonyl)amino) benzoate

Space Group:	P-1	***Cell:***	***a***	8.449(0)	***b***	8.951(0)	***c***	12.903(0)
Space Group No.:	2	***(Å,°)***	α	92.36(0)	β	104.60(0)	γ	92.58(0)
R-Factor (%):	4.90	***Temperature(K):***	173	***Density(g/cm³):***	1.391			

Parameters

Fragment 1	
**N3O2 (D)**	2.752
**NHO (A)**	140.017
**H3O2 (D)**	1.989
**N3H3 (D)**	0.913

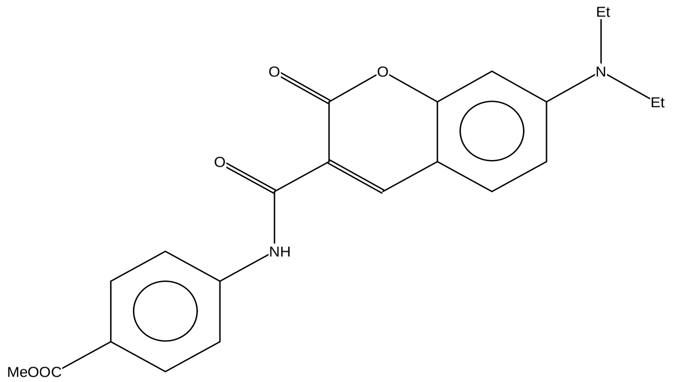

DISYEL

Reference:	M.Maldonado-Dominguez, R.Arcos-Ramos, M.Romero, B.Flores-Perez, N.Farfan, R.Santillan, P.G.Lacroix, I.Malfant (2014) *New J.Chem.* ,**38,**260
Formula:	$C_{21} H_{19} N_3 O_3$
Compound Name:	N-(4-Cyanophenyl)-7-(diethylamino)-2-oxo-2H-chromene-3-carboxamide

Space Group:	P-1	***Cell:***	***a***	6.903(0)	***b***	7.375(0)	***c***	19.537(0)
Space Group No.:	2	***(Å,°)***	α	81.88(0)	β	82.06(0)	γ	65.32(0)
R-Factor (%):	6.85	***Temperature(K):***	298	***Density(g/cm³):***	1.347			

Parameters

Fragment 1	
**N3O2 (D)**	2.701
**NHO (A)**	141.298
**H3O2 (D)**	1.898
**N3H3 (D)**	0.946

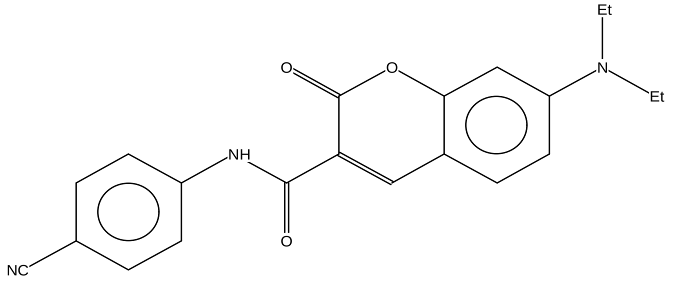

DISYIP

Reference: M.Maldonado-Dominguez, R.Arcos-Ramos, M.Romero, B.Flores-Perez, N.Farfan, R.Santillan, P.G.Lacroix, I.Malfant (2014) *New J.Chem.* ,**38,**260

Formula: $C_{20} H_{19} N_3 O_5$

Compound Name: 7-(Diethylamino)-N-(4-nitrophenyl)-2-oxo-2H-chromene-3-carboxamide

Space Group:	P-1	***Cell:***	***a*** 4.943(0)	***b*** 12.557(0)	***c*** 14.661(0)		
Space Group No.:	2	***(Å,°)***	α 98.17(0)	β 99.30(0)	γ 92.67(0)		
R-Factor (%):	4.82	***Temperature(K):***	298	***Density(g/cm³):***	1.429		

Parameters

Fragment 1

**N3O2 (D)**	2.722
**NHO (A)**	141.761
**H3O2 (D)**	1.995
**N3H3 (D)**	0.859

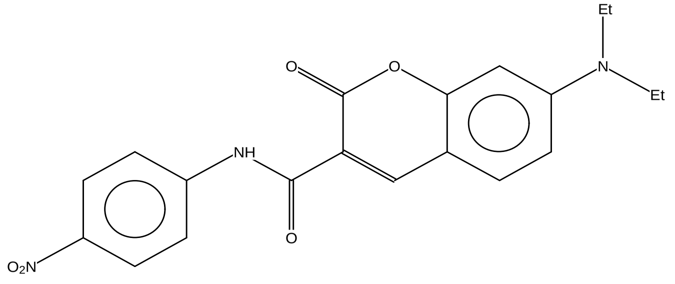

DUBJAN

Reference: Kangnan Wang, Zhiqiang Liu, Ruifang Guan, Duxia Cao, Hongyu Chen, Yanyan Shan, Qianqian Wu, Yongxiao Xu (2015) *Spectrochim.Acta,Part A* ,**144,**235

Formula: $C_{17} H_{10} N_2 O_3 S_1$

Compound Name: N-(1,3-Benzothiazol-2-yl)-2-oxo-2H-chromene-3-carboxamide

Space Group:	Pbn21	***Cell:***	***a*** 6.394(0)	***b*** 18.590(1)	***c*** 23.964(2)		
Space Group No.:	33	***(Å,°)***	α 90.00	β 90.00	γ 90.00		
R-Factor (%):	5.42	***Temperature(K):***	296	***Density(g/cm³):***	1.503		

Parameters

Fragment 1

**N3O2 (D)**	2.654
**NHO (A)**	141.644
**H3O2 (D)**	1.927
**N3H3 (D)**	0.858

Fragment 2

**N3O2 (D)**	2.641
**NHO (A)**	140.848
**H3O2 (D)**	1.917
**N3H3 (D)**	0.861

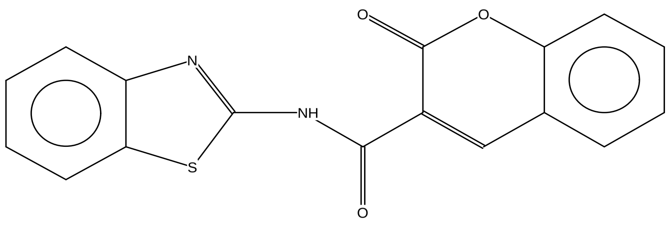

DUBJER

Reference:	Kangnan Wang, Zhiqiang Liu, Ruifang Guan, Duxia Cao, Hongyu Chen, Yanyan Shan, Qianqian Wu, Yongxiao Xu (2015) *Spectrochim.Acta,Part A* ,**144,**235
Formula:	C_{21} H_{19} N_3 O_3 S_1
Compound Name:	N-(1,3-Benzothiazol-2-yl)-7-(diethylamino)-2-oxo-2H-chromene-3-carboxamide

Space Group:	P21/n	***Cell:***	***a***	5.775(0)	***b***	17.554(0)	***c***	18.517(0)
Space Group No.:	14	***(Å,°)***	α	90.00	β	90.81(0)	γ	90.00
R-Factor (%):	6.39	***Temperature(K):***	296		***Density(g/cm³):***	1.392		

Parameters

Fragment 1	
**N3O2 (D)**	2.710
**NHO (A)**	138.439
**H3O2 (D)**	2.007
**N3H3 (D)**	0.859

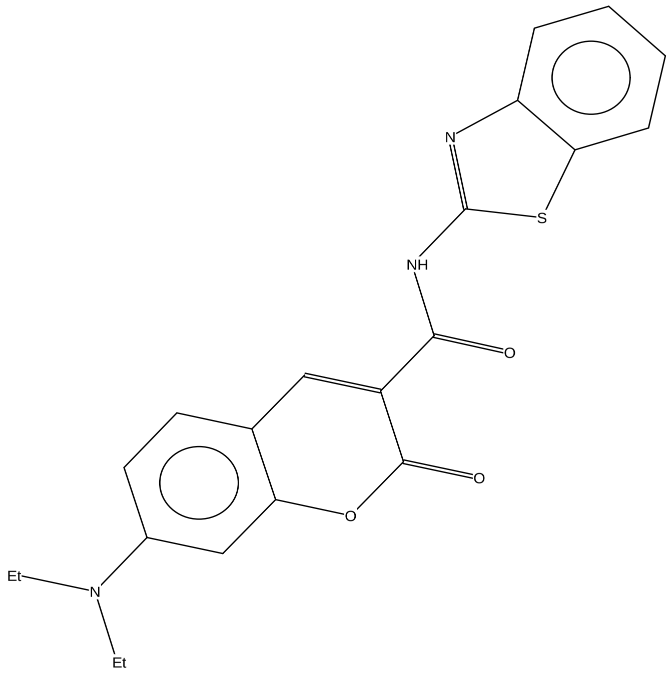

EFUVUY

Reference:	Xu He, Yan-Yan Chen, Jing-Bo Shi, Wen-Jiang Tang, Zhi-Xiang Pan, Zhi-Qiang Dong, Bao-An Song, Jun Li, Xin-Hua Liu (2014) *Bioorg.Med.Chem.* ,**22,**3732
Formula:	C_{14} H_{15} N_1 O_3
Compound Name:	N-t-butyl-2-oxo-2H-chromene-3-carboxamide

Space Group:	C2/c	***Cell:***	***a***	21.953(1)	***b***	8.869(0)	***c***	13.420(1)
Space Group No.:	15	***(Å,°)***	α	90.00	β	103.78(1)	γ	90.00
R-Factor (%):	7.16	***Temperature(K):***	293		***Density(g/cm³):***	1.284		

Parameters

Fragment 1	
**N3O2 (D)**	2.729
**NHO (A)**	145.034
**H3O2 (D)**	1.903
**N3H3 (D)**	0.942

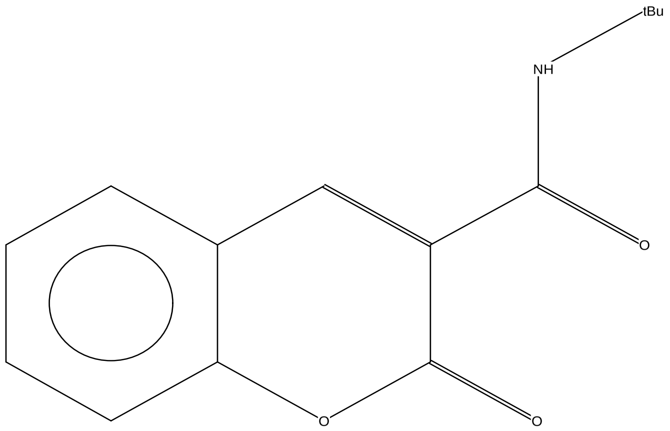

Search: search3 (Tue May 10 10:50:13 2016): Hits 11-12

GUCJEU

Reference: Guangjie He, Dong Guo, Cheng He, Xiaolin Zhang, Xiuwen Zhao, Chunying Duan (2009) *Angew.Chem.,Int.Ed.* ,**48,**6132

Formula: $C_{42}\ H_{45}\ N_5\ O_5,C_2\ H_6\ O_1$

Compound Name: N-(2-(3',6'-bis(Ethylamino)-2',7'-dimethyl-3-oxospiro[isoindole-1,9'-xanthen]-2(3H)-yl)ethyl)-7-(diethylamino)-2-oxo-2H-chromene-3-carboxamide ethanol solvate

Space Group:	P21/c	***Cell:***	***a*** 19.152(0)	***b*** 12.991(0)	***c*** 15.989(0)
Space Group No.:	14	***(Å,°)***	α 90.00	β 95.42(0)	γ 90.00
R-Factor (%):	5.85	***Temperature(K):*** 273		***Density(g/cm^3):*** 1.251	

Parameters

Fragment 1	
**N3O2 (D)**	2.703
**NHO (A)**	136.716
**H3O2 (D)**	2.012
**N3H3 (D)**	0.860

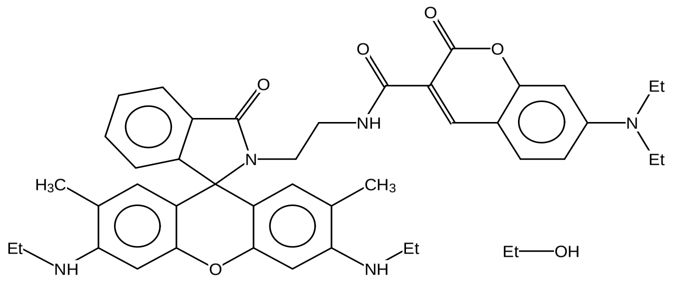

HAMVEX

Reference: N.E.Magana-Vergara, F.J.Martinez-Martinez, I.I.Padilla-Martinez, H.Hopfl, E.V.Garcia-Baez (2004) *Acta Crystallogr.,Sect.E:Struct.Rep.Online* ,**60,**o2306

Formula: $C_{16}\ H_{17}\ N_1\ O_3$

Compound Name: N-Cyclohexyl-2-oxo-2H-1-benzopyran-3-carboxamide

Space Group:	P-1	***Cell:***	***a*** 6.087(1)	***b*** 9.529(2)	***c*** 12.021(3)
Space Group No.:	2	***(Å,°)***	α 93.68(0)	β 96.98(0)	γ 103.54(0)
R-Factor (%):	5.60	***Temperature(K):*** 100		***Density(g/cm^3):*** 1.345	

Parameters

Fragment 1	
**N3O2 (D)**	2.743
**NHO (A)**	138.663
**H3O2 (D)**	1.986
**N3H3 (D)**	0.918

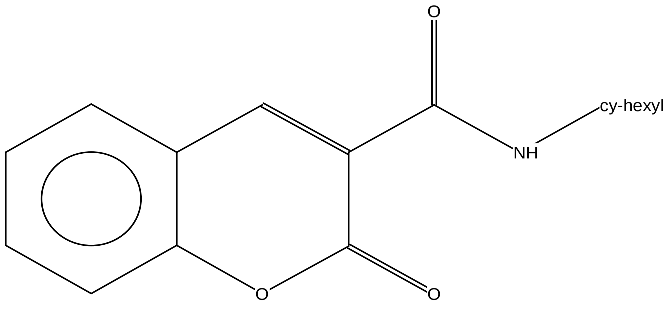

Search: search3 (Tue May 10 10:50:13 2016): Hits 13-14

LAGKEL

Reference: H.Kooijman, A.L.Spek, T.C.Chang, E.W.Meijer (2010)
CSD Communication(Private Communication) ,

Formula: $C_{27} H_{27} N_3 O_6$

Compound Name: N,N'-((Methylimino)dipropane-3,1-diyl)bis(2-oxo-2H-chromene-3-carboxamide)

**Space Group:**	P-1	**Cell:**	***a*** 8.818(1)	***b*** 10.390(1)	***c*** 13.700(2)
**Space Group No.:**	2	**(Å,°)**	α 108.90(1)	β 94.45(1)	γ 90.70(2)

R-Factor (%): 5.11 **Temperature(K):** 150 **Density(g/cm^3):** 1.374

Parameters

Fragment 1

**N3O2 (D)**	2.801
**NHO (A)**	124.690
**H3O2 (D)**	2.163
**N3H3 (D)**	0.934

Fragment 2

**N3O2 (D)**	2.744
**NHO (A)**	136.530
**H3O2 (D)**	2.016
**N3H3 (D)**	0.905

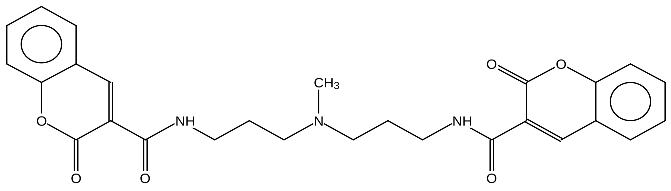

MUGQOW

Reference: Lingliang Long, Yanjun Wu, Lin Wang, Aihua Gong,
Feilong Hu, Chi Zhang (2015) *Chem.Commun.* ,**51,**10435

Formula: $C_{20} H_{21} N_3 O_3$

Compound Name: 7-(diethylamino)-2-oxo-N'-phenyl-2H-chromene-3-carbohydrazide

**Space Group:**	P21/c	**Cell:**	***a*** 13.155(0)	***b*** 6.940(0)	***c*** 21.079(1)
**Space Group No.:**	14	**(Å,°)**	α 90.00	β 109.63(0)	γ 90.00

R-Factor (%): 5.59 **Temperature(K):** 293 **Density(g/cm^3):** 1.288

Parameters

Fragment 1

**N3O2 (D)**	2.729
**NHO (A)**	135.630
**H3O2 (D)**	1.992
**N3H3 (D)**	0.924

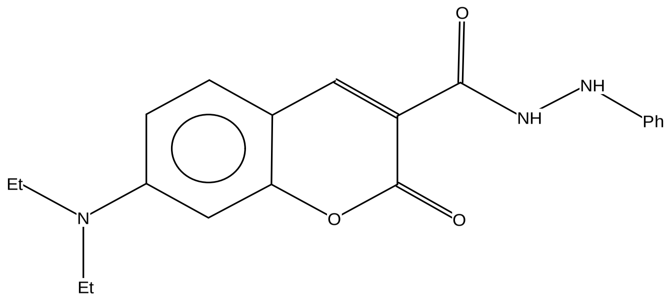

Search: search3 (Tue May 10 10:50:13 2016): Hits 15-16

MURDAE

Reference: E.V.Garcia-Baez, F.J.Martinez-Martinez, H.Hopfl, I.I.Padilla-Martinez (2003) *Cryst.Growth Des.* ,**3,**35

Formula: $C_{17} H_{13} N_1 O_3$

Compound Name: N-Benzyl-2-oxo-2H-1-benzopyran-3-carboxamide

Synonym: N-Benzylcoumarin-3-carboxamide

Space Group:	P-1	***Cell:***	***a*** 5.825(0)	***b*** 9.699(1)	***c*** 25.142(3)
Space Group No.:	2	***(Å,°)***	α 95.04(0)	β 94.26(0)	γ 101.52(0)

R-Factor (%): 4.67 ***Temperature(K):*** 293 ***Density(g/cm³):*** 1.344

Parameters

Fragment 1	
**N3O2 (D)**	2.704
**NHO (A)**	136.516
**H3O2 (D)**	1.873
**N3H3 (D)**	1.018

Fragment 2	
**N3O2 (D)**	2.713
**NHO (A)**	139.396
**H3O2 (D)**	1.893
**N3H3 (D)**	0.980

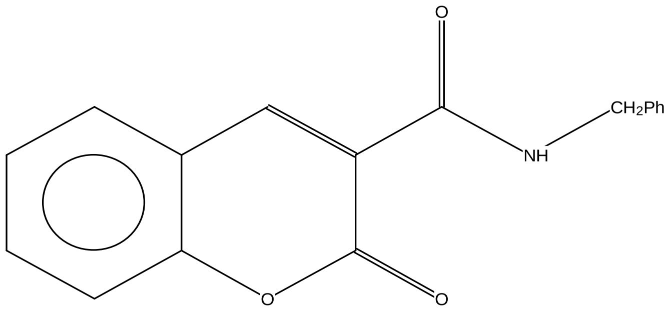

MURDEI

Reference: E.V.Garcia-Baez, F.J.Martinez-Martinez, H.Hopfl, I.I.Padilla-Martinez (2003) *Cryst.Growth Des.* ,**3,**35

Formula: $C_{18} H_{15} N_1 O_3$

Compound Name: N-(1-Phenylethyl)-2-oxo-2H-1-benzopyran-3-carboxamide

Synonym: N-(1-Phenylethyl)coumarin-3-carboxamide

Space Group:	P-1	***Cell:***	***a*** 5.891(0)	***b*** 7.996(0)	***c*** 16.114(2)
Space Group No.:	2	***(Å,°)***	α 98.32(0)	β 92.67(0)	γ 102.67(0)

R-Factor (%): 8.29 ***Temperature(K):*** 293 ***Density(g/cm³):*** 1.334

Parameters

Fragment 1	
**N3O2 (D)**	2.727
**NHO (A)**	132.775
**H3O2 (D)**	2.031
**N3H3 (D)**	0.904

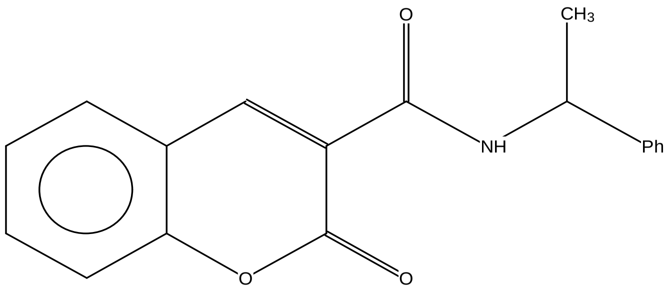

Search: search3 (Tue May 10 10:50:13 2016): Hits 17-18

NIHCUE

Reference: Xiaojing Wu, Hongda Li, Yuhe Kan, Bingzhu Yin (2013)
Dalton Trans. ,**42,**16302

Formula: $C_{28} H_{31} N_5 O_3$

Compound Name: N-(2-(bis(Pyridin-2-ylmethyl)amino)ethyl)-7-(diethylamino)-2-oxo-2H-chromene-3-carboxamide

Space Group:	P21/c	***Cell:***	***a*** 14.317(3) ***b*** 9.655(1) ***c*** 18.971(4)
Space Group No.:	14	***(Å,°)***	α 90.00 β 91.51(3) γ 90.00
R-Factor (%):	5.85	***Temperature(K):*** 291	***Density(g/cm³):*** 1.230

Parameters

Fragment 1	
**N3O2 (D)**	2.732
**NHO (A)**	137.563
**H3O2 (D)**	2.034
**N3H3 (D)**	0.860

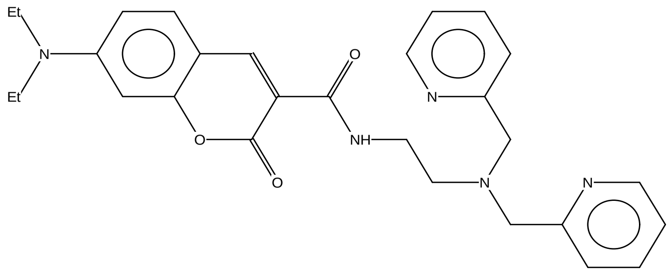

OJACAE

Reference: A.J.Clarke, N.Yamamoto, P.Jensen, T.W.Hambley (2009)
Dalton Trans. ,10787

Formula: $C_{16} H_{16} N_2 O_4$

Compound Name: N-Hydroxy-11-oxo-2,3,6,7-tetrahydro-1H,5H,11H-pyrano[2,3-f]pyrido[3,2,1-ij]quinoline-10-carboxamide

Space Group:	P21/c	***Cell:***	***a*** 12.032(0) ***b*** 8.449(0) ***c*** 13.564(0)
Space Group No.:	14	***(Å,°)***	α 90.00 β 105.45(0) γ 90.00
R-Factor (%):	4.30	***Temperature(K):*** 150	***Density(g/cm³):*** 1.501

Parameters

Fragment 1	
**N3O2 (D)**	2.670
**NHO (A)**	136.122
**H3O2 (D)**	1.974
**N3H3 (D)**	0.869

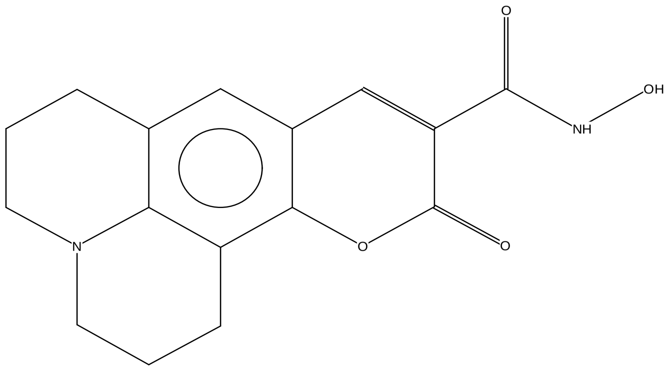

OKIREF

Reference: Lee-Chiang Lo, Jean-Yin Chen, Chun-Tzu Yang, Der-Shang Gu (2001) *Chirality* ,**13,**266

Formula: $C_{33} H_{37} N_3 O_9, C_6 H_{12}$

Compound Name: (2S,3R)-N,O-bis(7-Diethylaminocoumarin-3-carbonyl)threonine methyl ester cyclohexane solvate

Space Group:	P21	***Cell:***	***a*** 9.017(0)	***b*** 14.023(0)	***c*** 14.643(0)
Space Group No.:	4	***(Å,°)***	α 90.00	β 100.21	γ 90.00
R-Factor (%):	6.32	***Temperature(K):*** 150	***Density(g/cm³):*** 1.283		

Parameters

Fragment 1	
**N3O2 (D)**	2.706
**NHO (A)**	137.243
**H3O2 (D)**	1.993
**N3H3 (D)**	0.880

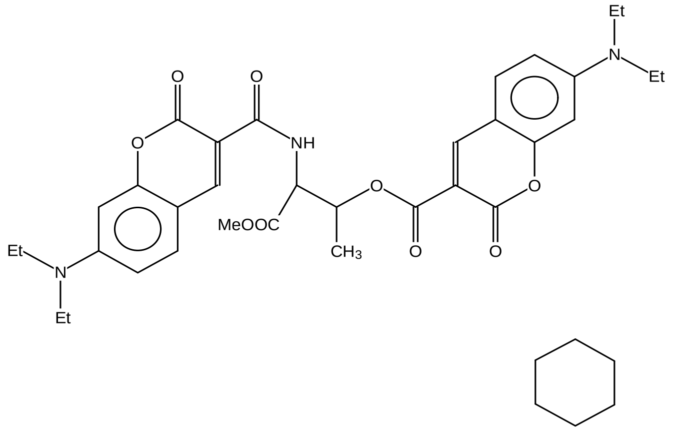

QUKBAA

Reference: Tianzhi Yu, Peng Zhang, Yuling Zhao, Hui Zhang, Jing Meng, Duowang Fan (2009) *Organic Electronics* ,**10,**653

Formula: $C_{18} H_{24} N_2 O_3$

Compound Name: N-Butyl-7-(diethylamino)-2-oxo-2H-chromene-3-carboxamide

Synonym: N-Butyl-7-(diethylamino)coumarin-3-carboxamide

Space Group:	C2/c	***Cell:***	***a*** 22.603(6)	***b*** 9.002(4)	***c*** 18.276(5)
Space Group No.:	15	***(Å,°)***	α 90.00	β 109.53(0)	γ 90.00
R-Factor (%):	10.37	***Temperature(K):*** 293	***Density(g/cm³):*** 1.199		

Parameters

Fragment 1	
**N3O2 (D)**	2.740
**NHO (A)**	136.183
**H3O2 (D)**	2.054
**N3H3 (D)**	0.860

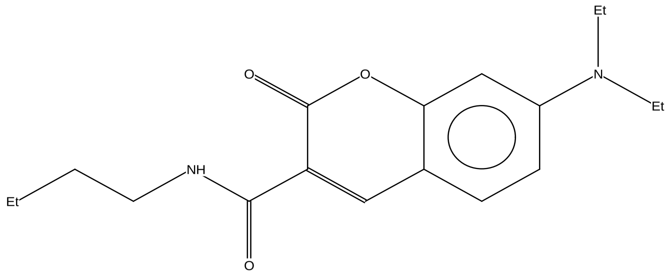

Search: search3 (Tue May 10 10:50:13 2016): Hits 21-22

RIRWOG

Reference: Da En, Yuan Guo, Bo-Ting Chen, Biao Dong, Meng-Jiao Peng (2015) *RSC Advances* ,**4,**248

Formula: $C_{14} H_{15} N_1 O_7$

Compound Name: N-(1,3-Dihydroxy-2-(hydroxymethyl)propan-2-yl)-7-hydroxy-2-oxo-2H-chromene-3-carboxamide

Space Group: P21/c
Space Group No.: 14

Cell: ***(Å,°)*** ***a*** 15.067(1) ***b*** 6.790(0) ***c*** 13.087(1) α 90.00 β 107.67(0) γ 90.00

R-Factor (%): 4.05 ***Temperature(K):*** 296 ***Density(g/cm³):*** 1.610

Parameters

Fragment 1

**N3O2 (D)**	2.707
**NHO (A)**	139.937
**H3O2 (D)**	1.992
**N3H3 (D)**	0.860

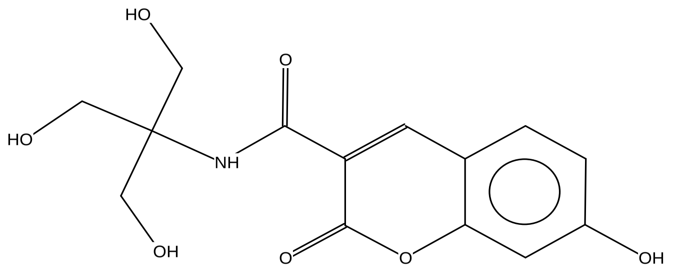

TASHAW

Reference: I.Cavaco, J.C.Pessoa, M.T.Duarte, R.D.Gillard, P.Matias (1996) *Chem.Commun.* ,1365

Formula: $C_{10} H_7 N_1 O_3$

Compound Name: Coumarin-3-carboxamide

Space Group: P21/c
Space Group No.: 14

Cell: ***(Å,°)*** ***a*** 4.764(2) ***b*** 14.387(8) ***c*** 12.379(2) α 90.00 β 95.75(2) γ 90.00

R-Factor (%): 6.40 ***Temperature(K):*** 295 ***Density(g/cm³):*** 1.488

Parameters

Fragment 1

**N3O2 (D)**	2.728
**NHO (A)**	140.659
**H3O2 (D)**	1.917
**N3H3 (D)**	0.960

Fragment 2

**N3O2 (D)**	2.728
**NHO (A)**	16.712
**H3O2 (D)**	3.691
**N3H3 (D)**	1.021

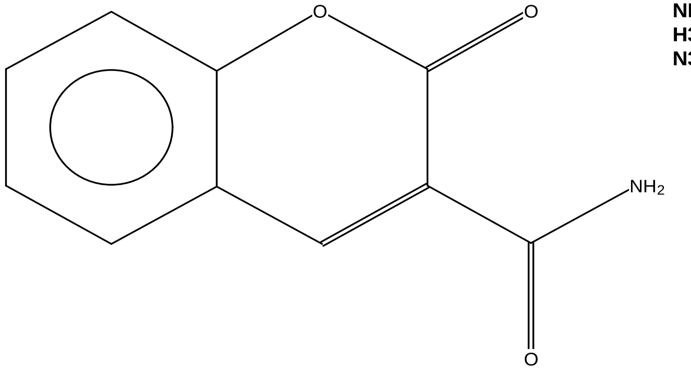

Search: search3 (Tue May 10 10:50:13 2016): Hits 23-24

TECDEL

Reference: Xiao-Feng Cheng, Li-Qiang Cao, Wen-Bin Chen, Hai-Bin Song (2006) *Acta Crystallogr.,Sect.E:Struct.Rep.Online* ,**62,** o721

Formula: $C_{15} H_{17} N_1 O_5 S_2$

Compound Name: N-(1-Hydroxy-3-(methylsulfanylmethylsulfinyl)propan-2-yl)-2-oxo-2H-chromene-3-carboxamide

Space Group:	P21	***Cell:***	***a***	10.323(1)	***b***	5.216(0)	***c***	15.448(3)
Space Group No.:	4	***(Å,°)***	α	90.00	β	98.34(0)	γ	90.00

R-Factor (%): 2.93 ***Temperature(K):*** 294 ***Density(g/cm^3):*** 1.434

Parameters

Fragment 1	
**N3O2 (D)**	2.719
**NHO (A)**	133.885
**H3O2 (D)**	2.120
**N3H3 (D)**	0.779

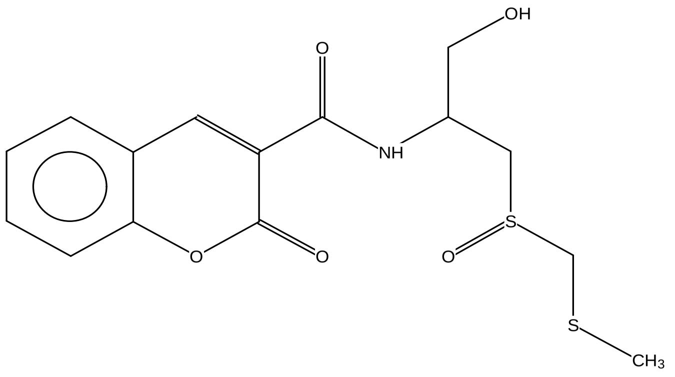

UHUVUP

Reference: R.J.Santos-Contreras, F.J.Martinez-Martinez, N.A.Mancilla-Margalli, A.L.Peraza-Campos, L.M.Morin-Sanchez, E.V.Garcia-Baez, I.I.Padilla-Martinez (2009) *CrystEngComm* ,**11,**1451

Formula: $C_{12} H_{11} N_1 O_4$

Compound Name: N-(2-Hydroxyethyl)-2-oxo-2H-chromene-3-carboxamide

Space Group:	P21/c	***Cell:***	***a***	17.372(4)	***b***	5.125(1)	***c***	11.968(3)
Space Group No.:	14	***(Å,°)***	α	90.00	β	103.92(0)	γ	90.00

R-Factor (%): 4.93 ***Temperature(K):*** 173 ***Density(g/cm^3):*** 1.498

Parameters

Fragment 1	
**N3O2 (D)**	2.721
**NHO (A)**	136.827
**H3O2 (D)**	2.011
**N3H3 (D)**	0.881

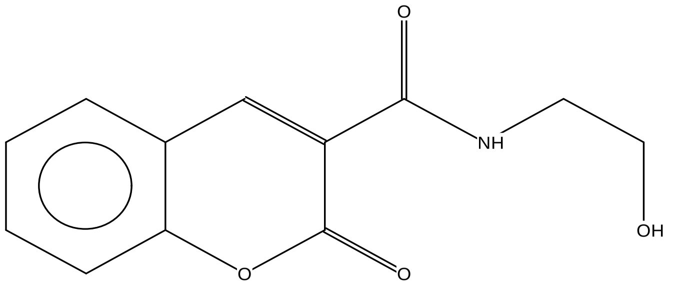

Search: search3 (Tue May 10 10:50:13 2016): Hits 25-26

UHUWAW

Reference: R.J.Santos-Contreras, F.J.Martinez-Martinez, N.A.Mancilla-Margalli, A.L.Peraza-Campos, L.M.Morin-Sanchez, E.V.Garcia-Baez, I.I.Padilla-Martinez (2009) *CrystEngComm* ,**11,**1451

Formula: $C_{12} H_{10} N_2 O_6 , H_2 O_1$

Compound Name: N-(2-Hydroxyethyl)-6-nitro-2-oxo-2H-chromene-3-carboxamide monohydrate

Space Group:	P-1	***Cell:***	***a*** 5.040(1)	***b*** 8.901(2)	***c*** 14.031(4)
Space Group No.:	2	***(Å,°)***	α 94.04(0)	β 91.53(0)	γ 90.09(0)
R-Factor (%):	5.37	***Temperature(K):***	293	***Density(g/cm³):***	1.568

Parameters

Fragment 1	
**N3O2 (D)**	2.740
**NHO (A)**	136.066
**H3O2 (D)**	2.038
**N3H3 (D)**	0.880

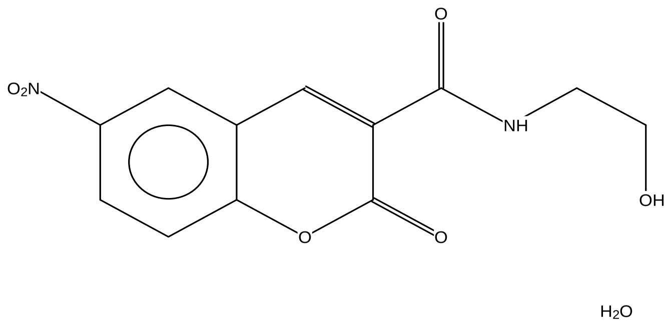

UHUWEA

Reference: R.J.Santos-Contreras, F.J.Martinez-Martinez, N.A.Mancilla-Margalli, A.L.Peraza-Campos, L.M.Morin-Sanchez, E.V.Garcia-Baez, I.I.Padilla-Martinez (2009) *CrystEngComm* ,**11,**1451

Formula: $C_{13} H_{13} N_1 O_5$

Compound Name: N-(2-Hydroxyethyl)-6-methoxy-2-oxo-2H-chromene-3-carboxamide

Space Group:	P21/c	***Cell:***	***a*** 6.805(1)	***b*** 24.947(5)	***c*** 8.097(3)
Space Group No.:	14	***(Å,°)***	α 90.00	β 123.45(2)	γ 90.00
R-Factor (%):	3.78	***Temperature(K):***	173	***Density(g/cm³):***	1.525

Parameters

Fragment 1	
**N3O2 (D)**	2.677
**NHO (A)**	137.950
**H3O2 (D)**	1.957
**N3H3 (D)**	0.881

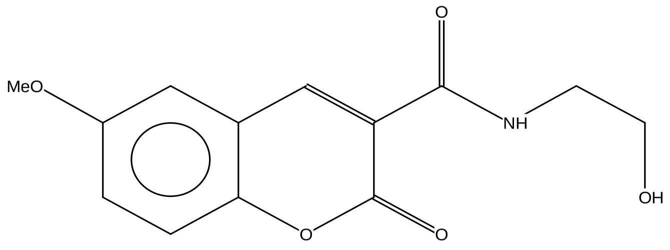

UHUWIE

Reference: R.J.Santos-Contreras, F.J.Martinez-Martinez, N.A.Mancilla-Margalli, A.L.Peraza-Campos, L.M.Morin-Sanchez, E.V.Garcia-Baez, I.I.Padilla-Martinez (2009) *CrystEngComm* ,**11,**1451

Formula: $C_{12}\ H_{10}\ Cl_1\ N_1\ O_4$

Compound Name: 6-Chloro-N-(2-hydroxyethyl)-2-oxo-2H-chromene-3-carboxamide

Space Group:	P21/c	***Cell:***	***a*** 5.357(0)	***b*** 22.078(2)	***c*** 9.309(0)		
Space Group No.:	14	***(Å,°)***	α 90.00	β 91.87(0)	γ 90.00		
R-Factor (%):	3.72	***Temperature(K):***	173	***Density(g/cm³):***	1.616		

Parameters

Fragment 1

**N3O2 (D)**	2.732
**NHO (A)**	137.746
**H3O2 (D)**	2.016
**N3H3 (D)**	0.879

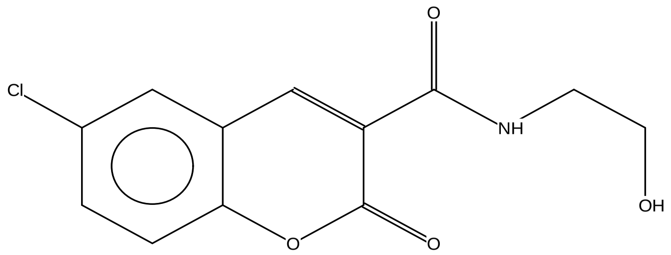

UHUWOK

Reference: R.J.Santos-Contreras, F.J.Martinez-Martinez, N.A.Mancilla-Margalli, A.L.Peraza-Campos, L.M.Morin-Sanchez, E.V.Garcia-Baez, I.I.Padilla-Martinez (2009) *CrystEngComm* ,**11,**1451

Formula: $C_{12}\ H_{10}\ Br_1\ N_1\ O_4$

Compound Name: 6-Bromo-N-(2-hydroxyethyl)-2-oxo-2H-chromene-3-carboxamide

Space Group:	P21/c	***Cell:***	***a*** 8.281(0)	***b*** 25.012(2)	***c*** 5.528(0)		
Space Group No.:	14	***(Å,°)***	α 90.00	β 94.46(0)	γ 90.00		
R-Factor (%):	5.44	***Temperature(K):***	173	***Density(g/cm³):***	1.816		

Parameters

Fragment 1

**N3O2 (D)**	2.729
**NHO (A)**	132.321
**H3O2 (D)**	2.058
**N3H3 (D)**	0.880

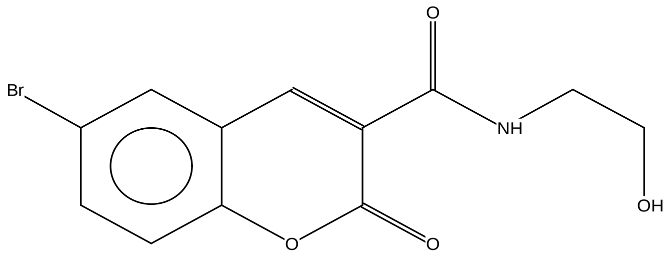

Search: search3 (Tue May 10 10:50:13 2016): Hits 29-30

URASIQ

Reference: Li-Jun Zhang, Bing-Zhu Yin (2011) *Acta Crystallogr.,Sect.E:Struct.Rep.Online* ,**67,**o1107

Formula: C_{14} H_{17} N_{3} O_{3}

Compound Name: 7-Diethylamino-2-oxo-2H-chromene-3-carbohydrazide

Space Group:	P-1	***Cell:***	***a*** 9.344(1)	***b*** 12.771(3)	***c*** 12.978(3)
Space Group No.:	2	***(Å,°)***	α 95.17(3)	β 110.13(3)	γ 106.18(3)

R-Factor (%): 4.47 ***Temperature(K):*** 290 ***Density(g/cm³):*** 1.338

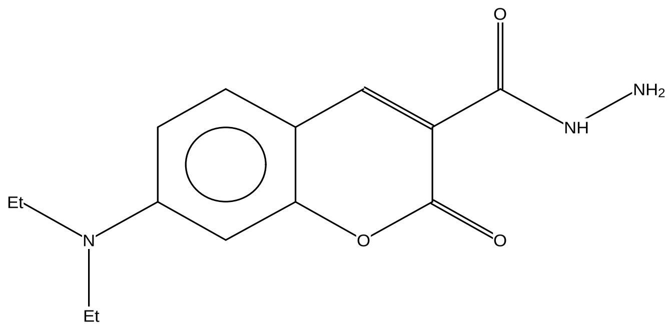

Parameters

Fragment 1	
**N3O2 (D)**	2.710
**NHO (A)**	137.263
**H3O2 (D)**	2.010
**N3H3 (D)**	0.865

Fragment 2	
**N3O2 (D)**	2.733
**NHO (A)**	138.012
**H3O2 (D)**	2.027
**N3H3 (D)**	0.866

URASIQ01

Reference: Lingliang Long, Yanjun Wu, Lin Wang, Aihua Gong, Feilong Hu, Chi Zhang (2015) *Chem.Commun.* ,**51,**10435

Formula: C_{14} H_{17} N_{3} O_{3}

Compound Name: 7-(diethylamino)-2-oxo-2H-chromene-3-carbohydrazide

Space Group:	P-1	***Cell:***	***a*** 9.212(1)	***b*** 12.624(3)	***c*** 12.927(3)
Space Group No.:	2	***(Å,°)***	α 94.78(3)	β 109.99(3)	γ 105.42(3)

R-Factor (%): 3.50 ***Temperature(K):*** 293 ***Density(g/cm³):*** 1.368

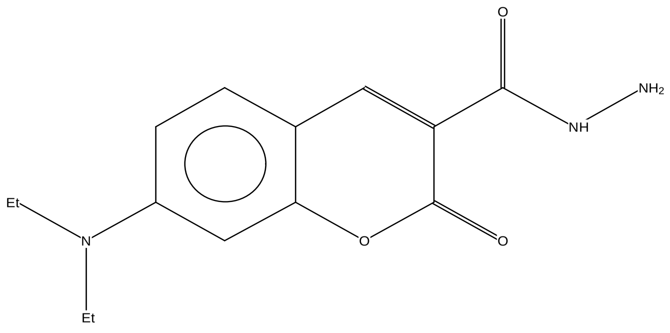

Parameters

Fragment 1	
**N3O2 (D)**	2.703
**NHO (A)**	138.404
**H3O2 (D)**	1.999
**N3H3 (D)**	0.860

Fragment 2	
**N3O2 (D)**	2.734
**NHO (A)**	136.731
**H3O2 (D)**	2.043
**N3H3 (D)**	0.860

WOJXOK

Reference: Zhi-Xiang Pan, Xu He, Yan-Yan Chen, Wen-Jian Tang, Jing-Bo Shi, Yiu-Lan Tang, Bao-An Song, Xin-Hua Liu (2014) *Eur.J.Med.Chem.* ,**80,**278

Formula: $C_{17} H_{11} Br_2 N_1 O_3$

Compound Name: 6,8-dibromo-N-(3-methylphenyl)-2-oxo-2H-chromene-3-carboxamide

Space Group:	I2/c	***Cell:***	***a***	16.632(3)	***b***	14.417(3)	***c***	13.258(3)
Space Group No.:	15	***(Å,°)***	α	90.00	β	95.66(1)	γ	90.00

R-Factor (%): 7.18 **_Temperature(K):_** 293 **_Density(g/cm^3):_** 1.835

Parameters

Fragment 1	
**N3O2 (D)**	2.761
**NHO (A)**	140.445
**H3O2 (D)**	2.043
**N3H3 (D)**	0.860

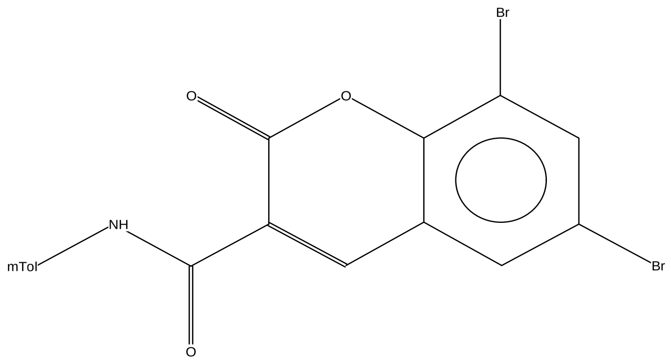

XEHDOF

Reference: E.Oliveira, C.Nunez, B.Rodriguez-Gonzalez, J.L.Capelo, C.Lodeiro (2011) *Inorg.Chem.* ,**50,**8797

Formula: $C_{14} H_{13} N_1 O_5 S_1$

Compound Name: methyl N-((2-oxo-2H-chromen-3-yl)carbonyl)cysteinate

Space Group:	P1	***Cell:***	***a***	5.098(0)	***b***	8.197(0)	***c***	8.537(0)
Space Group No.:	1	***(Å,°)***	α	99.25(0)	β	104.01(0)	γ	100.37(0)

R-Factor (%): 2.77 **_Temperature(K):_** 293 **_Density(g/cm^3):_** 1.535

Parameters

Fragment 1	
**N3O2 (D)**	2.733
**NHO (A)**	134.797
**H3O2 (D)**	2.006
**N3H3 (D)**	0.919

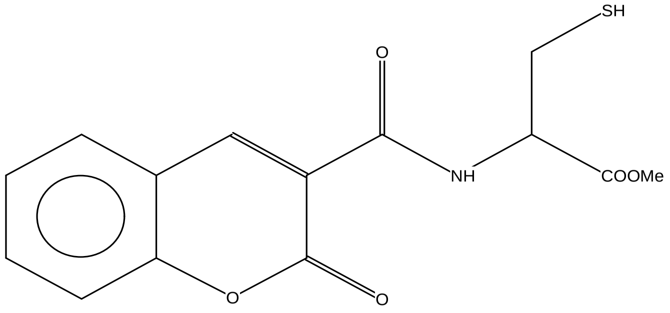

Search: search3 (Tue May 10 10:50:13 2016): Hits 33-34

ZOFWAU

Reference: Yan Bai, Ke Yu, Hui Pan, Dong-Bin Dang (2013) *Spectrochim.Acta,Part A* ,**113,**257

Formula: $C_{36}\ H_{38}\ N_4\ O_6$

Compound Name: N,N'-(1,3-Phenylenebis(methylene))bis(7-(diethylamino)-2-oxo-2H-chromene-3-carboxamide)

Space Group:	C2/c	***Cell:***	***a***	16.854(1)	***b***	7.038(0)	***c***	26.403(3)
Space Group No.:	15	***(Å,°)***	α	90.00	β	94.23(0)	γ	90.00

R-Factor (%): 5.65 ***Temperature(K):*** 296 ***Density(g/cm³):*** 1.324

Parameters

Fragment 1

**N3O2 (D)**	2.707
**NHO (A)**	138.804
**H3O2 (D)**	2.000
**N3H3 (D)**	0.860

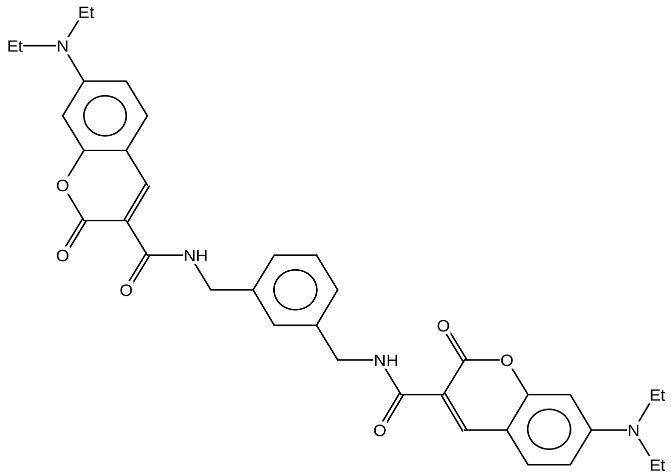

NABKOT

Reference: A.Saeed, S.Ashraf, U.Florke, Z.Y.D.Espinoza, M.F.Erben, H.Perez (2016) *J.Mol.Struct.* ,**1111,**76

Formula: $C_{18}\ H_{14}\ N_2\ O_4\ S_1$

Compound Name: N-((2-methoxyphenyl)carbamothioyl)-2-oxo-2H-chromene-3-carboxamide

Space Group:	P21/c	***Cell:***	***a***	7.455(2)	***b***	12.744(3)	***c***	16.892(4)
Space Group No.:	14	***(Å,°)***	α	90.00	β	90.20(0)	γ	90.00

R-Factor (%): 6.27 ***Temperature(K):*** 130 ***Density(g/cm³):*** 1.467

Parameters

Fragment 1

**N3O2 (D)**	2.721
**NHO (A)**	140.798
**H3O2 (D)**	1.981
**N3H3 (D)**	0.880

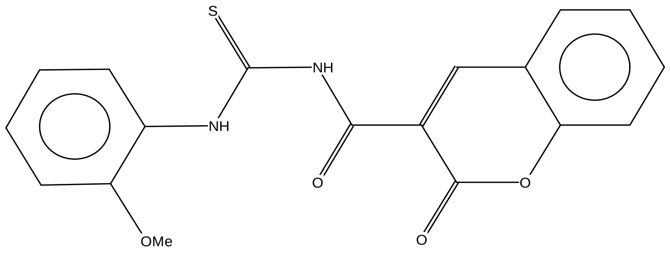

RUTTAD

Reference:	J.T.Mague, S.K.Mohamed, M.Akkurt, S.H.H.Younes, M.R.Albayati (2015) *Acta Crystallogr.,Sect.E:Cryst.Commun.* ,**71,**o1005
Formula:	$C_{16} H_{12} N_2 O_3$
Compound Name:	2-oxo-N'-phenyl-2H-chromene-3-carbohydrazide

Space Group:	P-1	***Cell:***	***a*** 6.651(0)	***b*** 8.391(0)	***c*** 11.639(0)		
Space Group No.:	2	***(Å,°)***	α 96.50(0)	β 95.61(0)	γ 94.76(0)		
R-Factor (%):	3.73	***Temperature(K):***	150	***Density(g/cm^3):***	1.456		

Parameters

Fragment 1

**N3O2 (D)**	2.754
**NHO (A)**	127.924
**H3O2 (D)**	2.109
**N3H3 (D)**	0.899

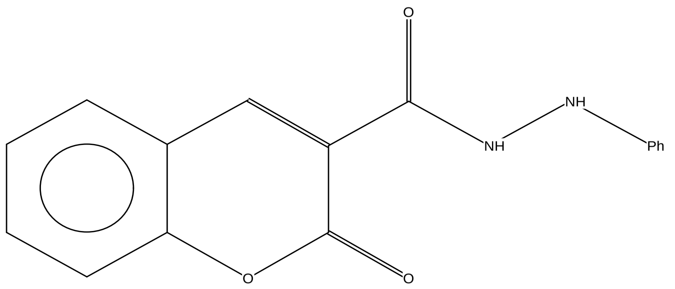

Supplement: Supplementary file 8 [file e-72-00926-sup8.pdf]
