# Supplementary material for: Crystal structures of three 6-substituted coumarin-3-carboxamide derivatives
Source: Acta Crystallogr E Crystallogr Commun. 2016 Jun 10;72(Pt 7):926–32. doi: 10.1107/S2056989016008665 (PMC4992908; doi:10.1107/S2056989016008665)

Search Overview

Search:	search2
Date/Time done:	Mon May 09 17:10:45 2016
Database(s):	CSD version 5.37 updates (Nov 2015)<br>CSD version 5.37 (November 2015)<br>CSD version 5.37 (November 2015)<br>CSD version 5.37 updates (Feb 2016)
Restriction Info:	No refcode restrictions applied
Filters:	3D coordinates determined<br>No errors<br>No ions<br>Only Organics<br>Not disordered<br>Not polymeric<br>No powder structures
Percentage Completed:	100%
Number of Hits:	6

Single query used. Search found structures that:

match

Query 1

Query 1

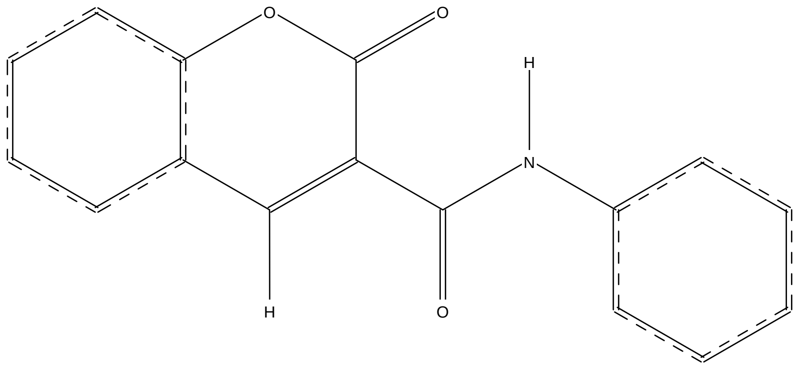

BONKAS

Reference: O.Julien, M.Kampmann, M.C.Bassik, J.A.Zorn, V.J.Venditto, K.Shimbo, N.J.Agard, K.Shimada, A.L.Rheingold, B.R.Stockwell, J.S.Weissman, J.A.Wells (2014) *Nat.Chem.Biol.* ,**10,**969

Formula: C_{24} H_{17} N_3 O_4

Compound Name: N-(3-(Imidazo[1,2-a]pyridin-2-yl)phenyl)-8-methoxy-2-oxo-2H-chromene-3-carboxamide

**_Space Group:_**	C2/c	**_Cell:_**	***a*** 36.299(4)	***b*** 6.374(0)	***c*** 18.828(2)
**_Space Group No.:_**	15	**_(Å,°)_**	α 90.00	β 121.05(0)	γ 90.00
**_R-Factor (%):_**	3.18	**_Temperature(K):_**	100	**_Density(g/cm³):_**	1.464

Parameters

Fragment 1

**CCN1 (T)**	-4.492
**CCN2 (T)**	177.134
**CNC1 (T)**	8.079
**CNC2 (T)**	-172.766

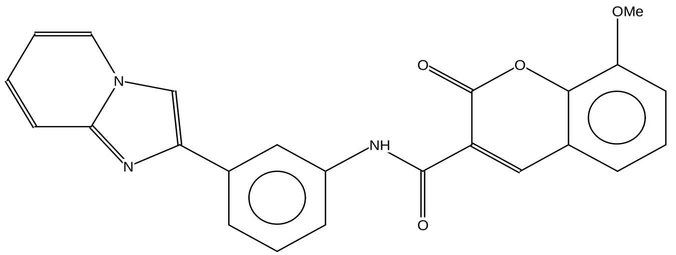

DISXUA

Reference: M.Maldonado-Dominguez, R.Arcos-Ramos, M.Romero, B.Flores-Perez, N.Farfan, R.Santillan, P.G.Lacroix, I.Malfant (2014) *New J.Chem.* ,**38,**260

Formula: C_{20} H_{19} F_1 N_2 O_3

Compound Name: 7-(Diethylamino)-N-(4-fluorophenyl)-2-oxo-2H-chromene-3-carboxamide

**_Space Group:_**	P-1	**_Cell:_**	***a*** 6.950(0)	***b*** 9.491(0)	***c*** 13.286(1)
**_Space Group No.:_**	2	**_(Å,°)_**	α 104.26(0)	β 91.62(0)	γ 100.78(0)
**_R-Factor (%):_**	4.93	**_Temperature(K):_**	132	**_Density(g/cm³):_**	1.415

Parameters

Fragment 1

**CCN1 (T)**	3.642
**CCN2 (T)**	-178.270
**CNC1 (T)**	-175.752
**CNC2 (T)**	4.910

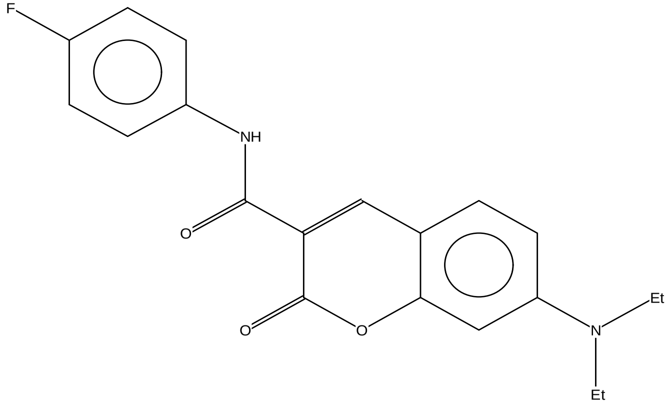

DISYAH

Reference: M.Maldonado-Dominguez, R.Arcos-Ramos, M.Romero, B.Flores-Perez, N.Farfan, R.Santillan, P.G.Lacroix, I.Malfant (2014) *New J.Chem.* ,**38,**260

Formula: C_{22} H_{22} N_2 O_5

Compound Name: Methyl 4-(((7-(diethylamino)-2-oxo-2H-chromen-3-yl)carbonyl)amino) benzoate

Space Group:	P-1	***Cell:***	***a***	8.449(0)	***b***	8.951(0)	***c***	12.903(0)
Space Group No.:	2	***(Å,°)***	α	92.36(0)	β	104.60(0)	γ	92.58(0)

R-Factor (%): 4.90 ***Temperature(K):*** 173 ***Density(g/cm³):*** 1.391

Parameters

Fragment 1	
**CCN1 (T)**	-2.311
**CCN2 (T)**	178.185
**CNC1 (T)**	-177.222
**CNC2 (T)**	3.158

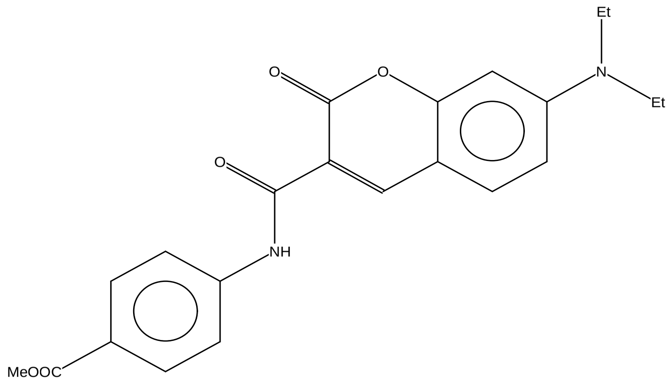

DISYEL

Reference: M.Maldonado-Dominguez, R.Arcos-Ramos, M.Romero, B.Flores-Perez, N.Farfan, R.Santillan, P.G.Lacroix, I.Malfant (2014) *New J.Chem.* ,**38,**260

Formula: C_{21} H_{19} N_3 O_3

Compound Name: N-(4-Cyanophenyl)-7-(diethylamino)-2-oxo-2H-chromene-3-carboxamide

Space Group:	P-1	***Cell:***	***a***	6.903(0)	***b***	7.375(0)	***c***	19.537(0)
Space Group No.:	2	***(Å,°)***	α	81.88(0)	β	82.06(0)	γ	65.32(0)

R-Factor (%): 6.85 ***Temperature(K):*** 298 ***Density(g/cm³):*** 1.347

Parameters

Fragment 1	
**CCN1 (T)**	-3.352
**CCN2 (T)**	176.959
**CNC1 (T)**	179.818
**CNC2 (T)**	0.000

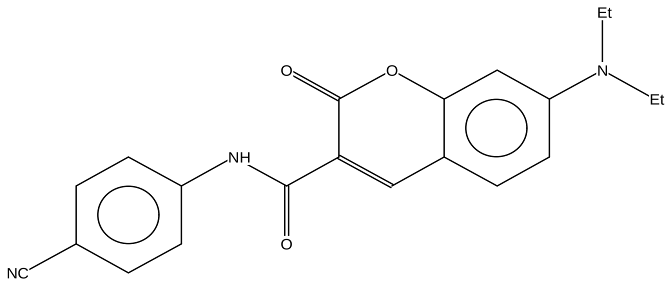

DISYIP

Reference: M.Maldonado-Dominguez, R.Arcos-Ramos, M.Romero, B.Flores-Perez, N.Farfan, R.Santillan, P.G.Lacroix, I.Malfant (2014) *New J.Chem.* ,**38,**260

Formula: $C_{20} H_{19} N_3 O_5$

Compound Name: 7-(Diethylamino)-N-(4-nitrophenyl)-2-oxo-2H-chromene-3-carboxamide

Space Group:	P-1	***Cell:***	***a***	4.943(0)	***b***	12.557(0)	***c***	14.661(0)
Space Group No.:	2	***(Å,°)***	α	98.17(0)	β	99.30(0)	γ	92.67(0)

R-Factor (%): 4.82 ***Temperature(K):*** 298 ***Density(g/cm³):*** 1.429

Parameters

Fragment 1

**CCN1**	**(T)**	-0.735
**CCN2**	**(T)**	-179.722
**CNC1**	**(T)**	-169.409
**CNC2**	**(T)**	11.066

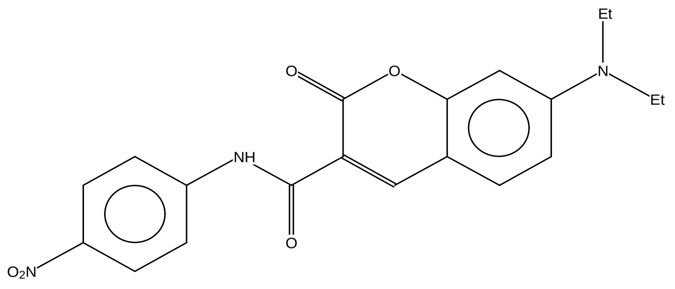

WOJXOK

Reference: Zhi-Xiang Pan, Xu He, Yan-Yan Chen, Wen-Jian Tang, Jing-Bo Shi, Yiu-Lan Tang, Bao-An Song, Xin-Hua Liu (2014) *Eur.J.Med.Chem.* ,**80,**278

Formula: $C_{17} H_{11} Br_2 N_1 O_3$

Compound Name: 6,8-dibromo-N-(3-methylphenyl)-2-oxo-2H-chromene-3-carboxamide

Space Group:	I2/c	***Cell:***	***a***	16.632(3)	***b***	14.417(3)	***c***	13.258(3)
Space Group No.:	15	***(Å,°)***	α	90.00	β	95.66(1)	γ	90.00

R-Factor (%): 7.18 ***Temperature(K):*** 293 ***Density(g/cm³):*** 1.835

Parameters

Fragment 1

**CCN1**	**(T)**	4.298
**CCN2**	**(T)**	-175.115
**CNC1**	**(T)**	-4.645
**CNC2**	**(T)**	177.530

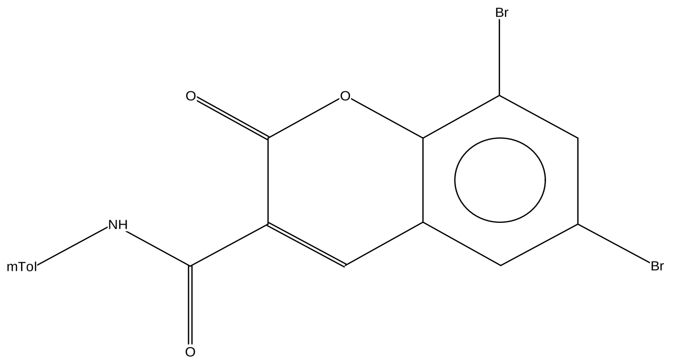

Supplement: Supplementary file 9 [file e-72-00926-sup9.pdf]
